# Supplementary material for: Integrative Analysis of Oleosin Genes Provides Insights into Lineage-Specific Family Evolution in Brassicales
Source: Plants (Basel). 2024 Jan 18;13(2):280. doi: 10.3390/plants13020280 (PMC10820149; doi:10.3390/plants13020280)
Supplement: Supplementary file 1 [file plants-13-00280-s001.zip › Figure S3.pdf]

Figure S3. The gene model of *CpOLE3*. The coding region is marked with uppercase letters, above which are its deduced amino acids (the oleosin domain is shown in red). The start and stop codons are marked with **bold** letters, whereas the conserved 12-residue proline knot is boxed.

```
  1 M  A  D  Q  P  H  Q  R  S  P  E  P  R  S  H  Q  V  V  K  A
  1 ATGGCTGATCAGCCCCACCAGCGCAGCCCTGAACCCCGCTCCCACCAGGTGGTCAAGGCC
 21 A  T  A  V  T  A  G  G  S  L  L  V  L  S  G  L  T  L  A  G
 61 GCCACCGCTGTCACTGCCGGTGGCTCCCTTTTGGTTCTCTCCGGTTTGACCCTAGCCGGT
 41 T  V  I  A  L  T  I  A  T  P  L  F  V  I  F  S  P  V  L  V
121 ACTGTCATTGCATTAACCATAGCTACGCCACTCTTCGTTATTTTCAGTCCGGTTCTTGTC
 61 P  A  V  I  A  V  A  L  L  I  T  G  F  L  A  S  G  G  F  G
181 CCTGCGGTTATCGCCGTTGCACTCTTGATCACGGGGTTCTTGGCTTCCGGTGGTTTTGGT
 81 V  A  A  I  T  V  L  S  W  I  Y  R
241 GTAGCTGCAATCACTGTCTTATCATGGATTTACAGgctagtactcatacatatataaata
301 taaatacgtgtacgtaaacatgtgtatattattcttaaaagttatttttataaacaaaat
 93             Y  V  T  G  R  H  P  P  G  A  D  K  L  D  Q  A  R
361 ggacatgcagGTATGTGACGGGAAGGCACCCACCGGGGGCGGACAAGCTGGACCAGGCAC
110   M  K  L  A  G  K  A  R  E  M  K  E  K  A  E  Q  Y  G  Q  Q
421 GCATGAAGTTAGCTGGAAAGGCTAGGGAGATGAAGGAGAAGGCGGAGCAGTACGGTCAGC
130   V  A  G  S  Q  G  S  *
481 AAGTGGCTGGGTCACAGGGTTCTTAA
```

Figure S3.2 The gene model of *MoOLE3*. The coding region is marked with uppercase letters, above which are its deduced amino acids (the oleosin domain is shown in red). The start and stop codons are marked with **bold** letters, whereas the conserved 12-residue proline knot is boxed.

1 M A E N L Q T K H R V T E P R Y R Q V V

1 **ATG**GCTGAGAACCTACAAACCAAGCATCGCGTCACTGAACCCAGGTATCGTCAGGTAGTC

21 K A A T A A T A G G S L L V L S G L T L

61 AAGGCGGCTACCGCAGCCACTGCTGGTGGATCCCTGTTGGTCCTCTCCGGTTTGACCCTT

41 A G T V I A L T I A T P L L V I F S P V

121 GCCGGCACGGTCATCGCACTGACCATCGCTACGCCGCTGCTGGTCATCTTCAGTCCGGTT

61 L V P A V I T V A L L I M G F L A S G G

181 CTAGTCCCTGCGGTGATCACTGTGGCACTCTTGATCATGGGGTTCTTGGCCTCGGGTGGT

81 F G V A S V T V L S W I Y R

241 TTTGGCGTTGCTTCAGTGACTGTGTTGTCATGGATCTACAGgtacgcacctcttctgttt

301 cctttaattaattagcgaaaattgaatcgaagttaacaatctgggtttgttaaacttaaa

95 Y V T G R H P V G A D Q L E Q A

361 ataacatgcagGTACGTGACTGGAAGGCACCCAGTAGGGGCGGACCAGCTGGAGCAAGCG

111 R M K L A S K A R E M K D K A S E Q F G

421 CGGATGAAGCTAGCCAGCAAGGCTCGAGAGATGAAGGATAAGGCGTCGGAGCAGTTCGGA

131 H Q V E I E R *

481 CACCAGGTGGAGATAGAGAGA**TGA**

Figure S3.3 The gene model of *BsOLE5*. The coding region is marked with uppercase letters, above which are its deduced amino acids (the oleosin domain is shown in red). The start and stop codons are marked with **bold** letters, whereas the conserved 12-residue proline knot is boxed.

```
  1 M  A  E  F  Y  Q  P  Q  Q  Q  S  Q  Q  T  R  S  H  N  V  V
  1 ATGGCCGAGTTTTACCAACCGCAGCAACAAAGCCAGCAGACGAGGTCCCACAACGTAGTT
 21 M  A  A  T  A  V  T  A  G  A  S  L  L  V  L  S  G  L  T  L
 61 ATGGCAGCCACTGCTGTCACCGCTGGTGCGTCTCTGCTGGTCCTCTCCGGTTTGACCCTC
 41 A  G  T  V  I  A  L  T  I  A  T  P  L  L  V  I  V  S  P  V
121 GCGGGCACGGTCATTGCACTGACCATAGCTACACCACTGCTGGTCATAGTCAGTCCGGTT
 61 L  V  P  A  V  I  T  V  G  L  L  I  M  G  F  L  T  S  G  G
181 CTTGTCCCTGCGGTGATCACAGTGGGTCTCTTGATAATGGGATTCCTGACCTCTGGTGGC
 81 F  G  V  A  A  V  T  V  V  S  W  I  Y  R
241 TTTGGTGTAGCTGCAGTTACCGTGGTATCATGGATCTACAGgtatcctacaaattaatag
301 agtaataattaataaaccagatcttttttttatatattttaaaaaacatattgcttcact
 95                                            Y  M  T  G  K  H  P  P  G
361 tgaactttctttttttaaaaattgacgtgtagGTATATGACTGGGAAGCACCCACCAGGG
104 A  D  R  L  D  S  A  R  M  K  L  A  S  K  A  R  E  M  K  D
421 GCGGACCGGCTGGACAGTGCACGCATGAAGCTAGCAAGCAAGGCTCGTGAGATGAAGGAC
124 K  A  E  Q  F  G  Q  Q  H  I  T  G  S  Q  A  S  *
481 AAGGCCGAGCAGTTTGGCCAGCAACACATCACTGGGTCACAAGCTTCTTAA
```

Figure S3.4 The gene model of *BsOLE6*. The coding region is marked with uppercase letters, above which are its deduced amino acids (the oleosin domain is shown in red). The start and stop codons are marked with **bold** letters, whereas the conserved 12-residue proline knot is boxed.

1 M A E Y H Q S Q Q P Q S H Q V V K A A T
1 **ATG**GCTGAGTATCACCAAAGCCAACAGCCCCAGTCCCACCAGGTAGTTAAGGCAGCCACT
21 A V T A G G S L L V L S G L T L A G T V
61 GCTGTCACCGCTGGTGGGTCCCTCCTGGTCCTCTCCGGTTTGACCCTGGCGGGTACGGTC
41 I A L T V A T P L L V I F S P V L V P A
121 ATTGCTCTGACCGTAGCTACACCACTGCTGGTCATATTCAGTCCGGTACTTGTCCCTGCG
61 V I T V G L L I M G F L T S G G F G V A
181 GTTATCACAGTGGGTCTCTTGATCATGGGGTTCTTGACCTCTGGTGGCTTCGGCGTAGCT
81 A V T V L S W I Y R
241 GCAGTTACCGTGCTATCATGGATCTACAGgtatcttacaaattataataattactactga
301 actagagagatttcttcttctttttaaaattatgttaaaaaattgctatacttggtcttt
91 Y V T G R H P P G
361 acttttttttttttttgaaattgacatgcagGTATGTGACTGGGAGGCACCCACCAGGG
100 A D Q L D S A R M K L A S K A R E M K E
421 GCGGACCAGCTGGACAGTGCACGCATGAAGCTAGCAAGTAAGGCTCGTGAGATGAAGGAA
120 K A E Q F G Q Q H I T G S Q A S *
481 AAAGCCGAGCAGTTTGGGCAGCAACACATCACTGGGTCACAAGCTTCT**TAA**

Figure S3.5 The gene model of *BsOLE7*. The coding region is marked with uppercase letters, above which are its deduced amino acids (the oleosin domain is shown in red). The start and stop codons are marked with **bold** letters, whereas the conserved 12-residue proline knot is boxed.

```
  1 M  A  E  Y  H  Q  S  Q  Q  P  Q  S  H  Q  V  V  K  A  A  T
  1 ATGGCTGAGTATCACCAAAGCCAACAGCCCCAGTCCCACCAGGTAGTTAAGGCAGCCACT
 21 A  V  T  A  G  G  S  L  L  V  L  S  G  L  T  L  A  G  T  V
 61 GCTGTCACCGCTGGTGGGTCCCTCCTGGTCCTCTCCGGTTTGACCCTGGCGGGTACGGTC
 41 I  A  L  T  V  A  T  P  L  L  V  I  F  S  P  V  L  V  P  A
121 ATTGCTCTGACCGTAGCTACACCACTGCTGGTCATATTCAGTCCTGTACTTGTCCCTGCG
 61 V  I  T  V  G  L  L  I  V  G  F  L  T  S  G  G  F  G  V  A
181 GTTATCACAGTGGGTCTCTTGATCGTGGGGTTCTTGACCTCTGGTGGCTTCGGTGTAGCT
 81 A  V  T  V  L  S  W  I  Y  R
241 GCAGTTACCGTGCTATCATGGATCTACAGgtatcttacaaattataataattactaatga
301 actagagagatttcttcttcgttttaaaattatgttaaaaaaattgctacacttggtctt
 91                                  Y  V  T  G  R  H  P  P  G
361 tactttttttgttttttgaaattgacatgcagGTATGTGACTGGGAGGCACCCACCAGGG
100 A  D  Q  L  D  S  A  R  M  K  L  A  S  K  A  R  E  M  K  E
421 GCGGACCAGCTGGACAGTGCACGCATGAAGCTAGCAAGTAAGGCTCGTGAGATGAAGGAA
120 K  A  E  Q  F  G  Q  Q  H  I  T  G  S  Q  A  S  *
481 AAAGCCGAGCAGTTTGGGCAGCAACACATCACTGGGTCACAAGCTTCTTAA
```

Figure S3.6 The gene model of *CsOLE3*. The coding region is marked with uppercase letters, above which are its deduced amino acids (the oleosin domain is shown in red). The start and stop codons are marked with **bold** letters, whereas the conserved 12-residue proline knot is boxed.

```
  1 M  Q  A  E  S  R  D  Q  P  P  S  R  P  R  S  Q  Q  M  V  K
  1 ATGCAAGCCGAGAGCCGCGACCAACCGCCTTCGCGACCGCGGTCGCAGCAGATGGTCAAG
 21 A  A  T  A  V  T  A  G  G  S  L  L  I  L  S  G  L  T  L  A
 61 GCGGCAACTGCCGTCACGGCTGGTGGGTCTCTCTTGATTCTCTCTGGTTTGACACTTGCC
 41 G  T  V  I  A  L  T  I  A  T  P  L  L  V  I  F  S  P  I  L
121 GGCACGGTTATAGCCTTAACCATCGCCACGCCGCTTTTGGTCATCTTCAGTCCGATTTTA
 61 V  P  A  V  I  T  V  S  L  L  I  V  G  F  L  A  S  G  G  F
181 GTACCGGCTGTCATCACCGTCAGTCTCTTGATCGTGGGGTTCTTGGCCTCCGGCGGCTTC
 81 G  V  A  S  I  T  I  F  S  W  V  Y  R
241 GGAGTAGCCTCCATTACCATCTTTTCATGGGTCTATAGgtaaattaattaacttgtccaa
301 atcacctcaatcaatggtggcttataattaatatcatcttataattaaaagctttttatt
361 catattaatttatttgcaccaagaagctcaacgcgcatgtacttgcatgccgatctactg
 94               Y  V  T  G  R  K  P  P  V  S  G  K  L  D  T  A
421 ataaattgtgtagGTATGTGACTGGAAGGAAGCCACCGGTGTCGGGGAAGCTGGACACGG
110   R  L  K  L  A  A  *
481 CGCGGTTGAAGCTGGCCGCATAG
```

Figure S3.7 The gene model of *CsOLE4*. The coding region is marked with uppercase letters, above which are its deduced amino acids (the oleosin domain is shown in red). The start and stop codons are marked with **bold** letters, whereas the conserved 12-residue proline knot is boxed.

1 M A E Q A R L P Y Q Q E P S R E Q P K S

1 **ATG**GCTGAACAAGCTCGGCTTCCTTACCAACAAGAGCCAAGCCGCGAACAGCCAAAATCG

21 R Q M V K A A T A V T A G G S L L I L S

61 CGGCAGATGGTGAAGGCCGCCACCGCCGTCACCGCTGGCGGTTCCCTTCTGATACTCTCC

41 G L T L A G T V I A L T V A T P L L V I

121 GGCCTAACCCTCGCCGGAACTGTCATAGCTCTGACGGTGGCGACGCCGCTTCTCGTTATC

61 F S P V L V P A V I T V A L L I T G F L

181 TTCAGTCCAGTTCTCGTTCCTGCTGTTATCACCGTTGCACTACTCATCACTGGGTTTCTT

81 A S G G F G I A G I T V F S W I Y R

241 GCCTCCGGTGGCTTCGGTATCGCAGGCATTACCGTCTTCTCCTGGATTTACAGgtaataa

301 tatctttcgtcgtctaatctgaatgtgtgtatctgtgcataagtctgtgcgtcggaatac

361 tgaatgtgtttcgtgcggttttcgtcggatatatagaattatattcaatttgccgatcta

421 tttttggtcggaaattattgtttttcaacaagaatatttaacactcctgtatttccaact

481 tttggttgaggtgaaagagaccatctcatccaagtgctaatgatttattttaatttaact

541 aaacaattgtattttttttttttttttgagctgcaaatcatttatcaaatttggtaacta

601 agtattagttgtcagttagagaaacccataattggaaaactgcagtaaggtctaaaaatt

99 Y V T G

661 ggcaagatgggaaataaaaggaacaatatacgaatgaaaattaatgtagGTACGTGACGG

103 K H P P G A D R L D S A R M K L A S K A

721 GAAAGCACCCGCCGGGGGCGGATAGGCTGGACAGTGCGAGAATGAAGCTGGCGAGCAAAG

123 Q E M K D R A Q Q F G Q Q H G G A G G G

781 CACAGGAGATGAAAGACAGGGCTCAGCAGTTCGGACAGCAACACGGTGGCGCTGGTGGGG

143 G G G Q Q T Y *

841 GCGGCGGTGGTCAGCAGACTTAT**TAA**

Figure S3.8 The gene model of *CsOLE5*. The coding region is marked with uppercase letters, above which are its deduced amino acids (the oleosin domain is shown in red). The start and stop codons are marked with **bold** letters, whereas the conserved 12-residue proline knot is boxed.

```
  1 M  Q  S  E  S  R  D  Q  P  Q  S  R  P  W  S  Q  Q  M  V  K
  1 ATGCAATCTGAGAGCCGCGACCAACCGCAGTCGCGACCCTGGTCGCAGCAGATGGTCAAG
 21 A  A  T  A  V  T  A  G  G  S  L  L  I  L  S  G  L  T  L  A
 61 GCTGCCACCGCCGTCACAGCCGGTGGATCTCTCTTGATTCTCTCCGGTTTGACCCTTGCG
 41 G  T  V  I  A  L  T  I  A  T  P  L  L  V  I  F  S  P  I  L
121 GGTACTGTTATAGCCTTAACCATTGCCACGCCGCTTTTGGTCATTTTCAGTCCCATTTTA
 61 V  P  A  I  I  S  V  S  L  L  I  V  G  F  L  A  S  G  G  F
181 GTACCGGCGATCATCTCCGTCAGTCTCTTGATCGTGGGGTTCTTGGCCTCCGGCGGCTTC
 81 G  V  A  A  I  T  I  F  S  W  V  Y  R
241 GGAGTAGCTGCCATCACCATTTTCTCATGGGTCTATAGgtaattataatttaagtaactt
301 aattatcaaatctatatataaatttatttaaacgacccattaatacatatatcacaaata
 94                                      Y  V  T  G  R  K  P  P
361 attcattgcacacacatgtatacgtgtatatatagGTATGTAACCGGAAGGAAGCCACCG
102 G  S  V  K  L  D  T  A  R  L  K  L  A  G  K  A  R  E  M  A
421 GGATCGGTGAAGCTGGACACGGCGCGGTTGAAGCTAGCGGGGAAGGCTCGAGAGATGGCG
122 E  R  A  E  N  Y  G  Q  H  Q  Q  S  P  *
481 GAGCGGGCCGAGAACTATGGTCAGCATCAACAATCTCCTTGA
```

Figure S3.9 The gene model of *CsOLE6*. The coding region is marked with uppercase letters, above which are its deduced amino acids (the oleosin domain is shown in red). The start and stop codons are marked with **bold** letters, whereas the conserved 12-residue proline knot is boxed.

```
  1 M  A  E  R  D  R  D  R  Q  I  Q  A  M  P  Y  E  G  G  G  P
  1 ATGGCCGAACGTGACCGTGACCGACAGATTCAAGCTATGCCTTATGAAGGCGGTGGCCCC
 21 A  A  K  S  G  P  P  Q  R  G  P  S  S  S  Q
 61 GCCGCCAAGAGTGGACCTCCTCAACGCGGCCCTTCTAGCTCTCAGgcaatgttattatgg
121 ttattattattttgtttcaatgatgtgagaagatgtaatcattgtaagcaaaagtaactt
 36                   I  L  A  L  L  A  G  I  P  V  G  G  T  L
181 gatatatagggtgatgcagATCTTGGCCCTTTTGGCCGGTATCCCGGTTGGCGGTACGCT
 50  L  A  L  A  G  L  A  L  A  G  S  V  I  G  L  L  I  A  T  P
241 TCTAGCACTAGCCGGTCTAGCTTTAGCTGGCTCGGTGATAGGACTATTAATCGCGACGCC
 70  L  F  I  I  F  S  P  V  I  V  P  A  A  I  T  I  G  L  A  V
301 ACTCTTCATTATCTTCAGCCCAGTGATCGTTCCAGCGGCGATCACCATCGGGCTAGCGGT
 90  A  G  I  L  A  S  G  L  F  G  L  T  G  L  S  S  I  S  W  V
361 TGCGGGTATTCTAGCCTCCGGGCTTTTTGGGCTAACAGGCTTGAGTTCGATCTCGTGGGT
110  M  N  Y  L  R  G  A  Q  P  P  R  T  I  P  E  Q  I  E  Y  A
421 CATGAATTACCTCCGTGGAGCACAGCCCCCACGGACCATACCGGAGCAGATTGAGTACGC
130  K  R  R  M  G  E  V  V  E  Q  A  G  Q  K  A  K  E  M  S  H
481 CAAGAGAAGGATGGGCGAGGTGGTCGAACAGGCGGGCCAGAAGGCGAAAGAGATGAGCCA
150  *
541 CTAG
```

(Boxed proline knot: G L L I A T P at residues 63–69 and L F I I F S P at residues 70–76.)

Figure S3.10 The gene model of *CsOLE7*. The coding region is marked with uppercase letters, above which are its deduced amino acids (the oleosin domain is shown in red). The start and stop codons are marked with **bold** letters, whereas the conserved 12-residue proline knot is boxed.

```
  1 M  A  D  R  D  R  D  R  Q  I  Q  A  Y  E  G  A  G  P  G  G
  1 ATGGCCGACCGCGACCGTGACCGTCAGATTCAAGCTTATGAAGGCGCCGGCCCCGGCGGC
 21 K  T  E  P  S  Q  R  G  P  S  S  S  Q
 61 AAGACCGAACCCTCTCAACGCGGCCCCTCTAGCTCTCAGgttaacattattaagttaaaa
121 gatagagaattaggtagaattaagtgatcgattgataaattataaaatatgggtatattt
 34                                I  V  A  L  L  T  G  I  P  V  G
181 tgcgaaaatttattttctgacaacgcagATCGTGGCCCTTTTGACCGGGATTCCGGTTGG
 45 G  T  L  L  A  L  A  G  L  A  L  A  G  S  V  V  G  L  L  I
241 AGGGACTCTCCTGGCCTTAGCCGGTCTAGCTCTAGCAGGCTCGGTGGTCGGCCTATTAAT
 65 A  T [P  L  F  I  I  F  S  P  V  I  V  P] A  A  I  T  I  G
301 CGCAACGCCGCTTTTCATCATCTTCAGCCCGGTGATCGTCCCGGCGGCGATCACGATCGG
 85 L  A  V  A  G  F  L  A  S  G  L  F  G  L  T  G  L  S  S  I
361 GTTAGCAGTAGCGGGTTTTCTAGCGTCGGGGCTATTCGGGCTAACGGGCCTGAGTTCGAT
105 S  W  V  M  N  Y  L  R  G  V  Q  P  Q  T  Q  R  T  I  P  E
421 CTCGTGGGTGATGAACTACCTCCGTGGAGTCCAACCCCAAACCCAACGGACCATACCGGA
125 Q  I  D  Y  A  K  R  R  M  G  E  M  I  G  H  A  G  Q  K  G
481 GCAGATTGACTATGCAAAGCGACGTATGGGAGAGATGATTGGACATGCGGGCCAGAAGGG
145 K  E  M  G  Q  Q  I  Q  N  K  G  Q  E  A  Q  K  T  *
541 GAAGGAAATGGGCCAGCAGATCCAGAATAAGGGCCAGGAGGCCCAGAAGACATGA
```

Figure S3.11 The gene model of *CsOLE8*. The coding region is marked with uppercase letters, above which are its deduced amino acids (the oleosin domain is shown in red). The start and stop codons are marked with **bold** letters, whereas the conserved 12-residue proline knot is boxed.

1 M A E V R T Q P H Q L Q V H P Q R H E E

1 **ATG**GCGGAAGTCCGTACCCAACCGCACCAGCTTCAAGTTCACCCGCAACGCCATGAGGAA

21 G Q R R P S A I Q

61 GGCCAGCGCCGGCCATCTGCTATTCAGgtctttttttcccctctctctatatatctacac

121 ggtttcgtgtatatgtatatatatggatcgaagacaaaagtaacttaggttatgcatgtg

30 V L A V V T G L P V G G T L L T L A

181 gtgacagGTGCTAGCGGTGGTGACAGGGTTACCAGTCGGAGGAACACTGCTAACCTTGGC

48 G L T L A G S V I G L M V A F P L F I I

241 CGGTTTGACGCTAGCCGGTTCAGTTATCGGGCTCATGGTCGCCTTTCCGTTGTTCATAAT

68 F S P V I V P A A F V I A L A V T G F F

301 CTTCAGCCCAGTTATCGTGCCAGCAGCCTTTGTGATTGCCCTCGCGGTTACTGGCTTCTT

88 V S G A F G L S G L S S M S W V M N Y L

361 CGTGTCAGGCGCGTTTGGGCTCTCGGGCTTGTCGTCGATGTCATGGGTAATGAACTATCT

108 R Q V K E S T P D Q L E L A K K R M A D

421 ACGACAGGTGAAGGAGTCAACGCCAGATCAGCTCGAACTGGCAAAGAAGCGCATGGCGGA

128 M A G Y V G Q K T K E A G Q K I E S K A

481 CATGGCCGGGTATGTGGGACAGAAGACAAAAGAAGCCGGACAAAAGATAGAGTCTAAGGC

148 H E S R T *

541 TCATGAATCTAGGACA**TGA**

Figure S3.12 The gene model of *CvOLE1*. The coding region is marked with uppercase letters, above which are its deduced amino acids (the oleosin domain is shown in red). The start and stop codons are marked with **bold** letters, whereas the conserved 12-residue proline knot is boxed.

1 M A D R N P N T Q R P M Y G P H G G S R

1 **ATG**GCCGATCGAAACCCTAACACGCAGAGACCCATGTACGGTCCTCACGGCGGCAGCCGC

21 P V A A L L R Q L Q S H A P T S A Q L F

61 CCCGTCGCCGCCCTCCTCCGCCAGCTCCAATCCCACGCGCCCACCTCCGCCCAGCTCTTC

41 G F L T L F I A G G I L L F L T G V T V

121 GGCTTCCTCACCCTCTTCATCGCCGGCGGAATCCTCCTCTTCCTCACCGGCGTCACCGTC

61 T A A I L G F I A F L P L I L L L S P I

181 ACCGCCGCCATCCTCGGCTTCATCGCTTTCCTCCCCCTCATCCTCCTCTTGAGCCCAATC

81 W

241 TGgtaatttcacatccatcattatgcacgggatccagattcatgcatgaaccactcgacc

301 acggatctttttttgttcttttgaatacgtatgatccatgcgtatcatttaatatggacgt

361 atcccaaaacaatggtttcgggaatccagatcgtgaaccacttgaacacgggcctttgtt

421 tgttcctttgaatacgtatgattcaatcaggcttctaaaaatcggctttaggcggtgtct

481 aggcgttacgcagccgccaggtgccgcgaggaacgaaaaatcggcgaagaaatcggtttt

541 taattaatcggtcgttttctcgattaatcagcgtctaggcagcgattaaattaatcggtc

601 aattaatcggaaaatcgtcgtctagggaccaattaatcggaaaaaatatgtttttttctt

661 gtacctcacaaaacaacgttgttttgggtagattccggcaaaaaaaaaaggaaaaaaaaa

721 atttaaacaccggctagacggcggtccgacggtgcataacggtttttataacattgatat

82 V P A V V L M G G F L T V A G

781 gaattaattttgacagGGTTCCCGCGGTGGTACTCATGGGCGGGTTCTTGACGGTGGCGG

97 S T I A T A A V V S W T Y R Y L R G M H

841 GATCGACCATCGCGACAGCGGCGGTCGTGTCGTGGACGTACCGGTATCTCCGGGGCATGC

117 P V G S D Q V D Y A R T R I Y D T A A H

901 ACCCCGTCGGGTCGGACCAGGTGGATTACGCCAGGACTCGGATCTACGACACGGCGGCTC

137 V K D Y A R E Y G G Y L Q S K V K D A A

961 ACGTCAAAGACTACGCCAGGGAGTACGGTGGTTACCTCCAAAGCAAGGTTAAGGATGCGG

157 P G A *

1021 CGCCTGGTGCG**TGA**

Figure S3.13 The gene model of *CvOLE3*. The coding region is marked with uppercase letters, above which are its deduced amino acids (the oleosin domain is shown in red). The start and stop codons are marked with **bold** letters, whereas the conserved 12-residue proline knot is boxed.

```
  1 M  A  D  V  S  S  R  P  H  D  M  S  R  D  Q  P  K  S  R  Q
  1 ATGGCAGACGTCTCATCTCGACCGCACGATATGAGCCGAGATCAGCCCAAGTCGAGGCAG
 21 M  V  K  A  A  T  A  V  T  A  G  G  S  L  L  V  L  S  G  L
 61 ATGGTGAAGGCGGCGACTGCCGTGACCGCTGGTGGGTCCCTCCTGGTTCTCTCCGGCTTG
 41 T  L  A  G  T  V  I  A  L  T  V  A  T  P  L  L  V  I  F  S
121 ACCCTCGCCGGAACTGTGATCGCTCTCACGGTGGCGACTCCGCTTCTCGTCATCTTCAGC
 61 P  V  L  V  P  A  V  I  T  I  A  L  L  I  T  G  F  L  A  S
181 CCGGTGCTCGTCCCCGCTGTCATCACCATAGCTCTGCTCATCACCGGCTTCCTTGCCTCC
 81 G  G  F  G  I  A  G  I  T  V  F  S  W  I  Y  R
241 GGAGGCTTCGGGATCGCCGGCATTACCGTCTTCTCTTGGATTTACAGgtaacaatacaca
301 cgcacagggcatatatgtgtaagtattgaagggtggagaatacatatgtatatgtgtgtg
 97             Y  V  T  G  R  H  P  M  G  A  D  K  L  D  S  A  R
361 tatgcgtagGTACGTGACGGGGAGGCATCCGATGGGGGCGGACAAGCTGGACAGCGCGAG
114  M  K  L  A  S  K  A  Q  E  M  K  D  R  A  Q  Q  F  G  Q  Q
421 GATGAAGCTGGCGAGCAAAGCTCAGGAGATGAAAGACAGAGCTCAGCAGTTCGGACAGCA
134  H  S  G  G  G  T  H  Q  T  S  *
481 ACACTCTGGCGGCGGAACCCACCAGACTTCTTAG
```

Figure S3.14 The gene model of *CvOLE4*. The coding region is marked with uppercase letters, above which are its deduced amino acids (the oleosin domain is shown in red). The start and stop codons are marked with **bold** letters, whereas the conserved 12-residue proline knot is boxed.

```
  1 M  A  D  R  D  R  D  R  Q  V  H  P  Y  E  G  G  G  Q  G  I
  1 ATGGCGGATCGGGACCGTGATCGGCAAGTTCATCCGTACGAAGGCGGAGGCCAAGGCATC
 21 K  S  I  V  P  D  R  G  P  S  S  T  Q
 61 AAGAGCATCGTCCCTGACCGAGGCCCCTCCAGTACCCAGgtttatatatatatacacaca
121 ttacgtgattcgcgatatttaaatttcactatatatatcgatcttgcaatatgtatacat
181 gtatatatcatatttatacaccttgttaggcaaaaccaacggtttcaaacaatgtccaaa
241 ttttatttacgcgggtgggtgttaatttcacgagctatgactcggtttatatatcgtgga
301 ccgacgagggttcgatgaaatttcattaaatgtcttcttggttacggcctcagattctta
361 tgttagcgtctgaatatgtatgtatgatgcagctgtcagattcatatcgtttttctcata
 34                             V  L  A  L  L  T  G  V  P  I  G
421 tgtgtataaatataaccgatgcatgcagGTGTTGGCTCTCTTGACTGGTGTCCCGATCGG
 45  G  T  L  L  A  L  A  G  L  A  L  A  G  S  V  I  G  L  M  I
481 CGGGACGCTGCTGGCCCTAGCCGGCTTGGCTCTGGCAGGCTCGGTGATCGGCTTGATGAT
 65  A  T [P  I  F  I  I  F  S  P  V  I  V  P] A  A  I  T  V  G
541 AGCGACGCCGATCTTCATCATCTTCAGCCCGGTGATAGTCCCCGCGGCAATCACGGTCGG
 85  L  A  V  A  G  I  L  S  S  G  L  F  G  L  T  G  L  S  S  I
601 GCTAGCGGTCGCAGGCATCCTGTCGTCGGGACTGTTCGGGCTGACGGGGCTGAGCTCGAT
105  S  W  V  M  N  Y  L  R  G  T  R  R  S  M  P  E  Q  M  D  Y
661 CTCGTGGGTCATGAACTACCTCCGTGGCACGAGACGGTCCATGCCCGAGCAGATGGACTA
125  A  K  R  R  M  A  D  V  V  G  F  A  G  Q  K  T  K  D  V  G
721 CGCCAAGCGTAGGATGGCCGACGTGGTGGGCTTTGCGGGCCAGAAGACCAAGGACGTGGG
145  Q  T  V  Q  S  K  A  H  E  V  S  R  T  *
781 CCAGACCGTGCAGAGCAAGGCCCATGAGGTCTCCAGGACTTGA
```

Figure S3.15 The gene model of *CvOLE5*. The coding region is marked with uppercase letters, above which are its deduced amino acids (the oleosin domain is shown in red). The start and stop codons are marked with **bold** letters, whereas the conserved 12-residue proline knot is boxed.

1 M A D V R T Q A H Q L Q V H P Q R Q H E
1 **ATG**GCAGACGTCCGTACACAAGCCCACCAACTCCAAGTTCATCCACAACGCCAACATGAA
21 G G M K T L L P Q R G P S A T Q
61 GGGGGCATGAAGACCCTCCTTCCCCAACGCGGCCCTTCTGCTACACAGgtgactatttct
121 tttctttttgtaataatacatgtacgagctcccacatacatatagctaatatgtataatg
37 V L
181 cagtttacatatctggggtttaatttgttacatggcgacacggttggcatgcagGTACTG
39 A V V T G I P V G G T L M A L A G L T L
241 GCAGTGGTGACTGGCATACCGGTCGGAGGCACATTGATGGCCTTGGCCGGTTTGACACTG
59 A G S V I G L M V A F P L F V I F S P I
301 GCTGGTTCAGTTATCGGGCTCATGGTGGCATTTCCATTGTTTGTGATCTTCAGTCCGATA
79 I V P A A I A I G L A V M G I L A S G A
361 ATCGTCCCAGCCGCCATAGCTATTGGTCTGGCAGTTATGGGGATTCTGGCGTCGGGAGCA
99 I G L T G L S S M S W V M N Y L V R A K
421 ATTGGGCTGACGGGGTTGTCGTCGATGTCCTGGGTCATGAACTATCTCGTCAGAGCGAAG
119 D A V P E Q L D Y A K R R M A G M A G Y
481 GATGCTGTACCAGAGCAGCTCGACTACGCCAAGCGCCGCATGGCTGGCATGGCCGGATAT
139 V G S K T K D M G Q S I E S K A H E V Q
541 GTGGGTTCAAAGACTAAAGATATGGGACAATCAATAGAGAGTAAGGCGCATGAAGTGCAG
159 V S T *
601 GTGTCGACA**TGA**

Figure S3.16 The gene model of *GgOLE1*. The coding region is marked with uppercase letters, above which are its deduced amino acids (the oleosin domain is shown in red). The start and stop codons are marked with **bold** letters, whereas the conserved 12-residue proline knot is boxed.

```
  1 M  A  D  Q  N  P  N  Q  R  P  I  Y  G  G  G  G  A  G  L  H
  1 ATGGCCGACCAAAACCCTAACCAGAGACCCATCTACGGCGGGGGAGGCGCGGGCTTGCAT
 21 H  G  G  G  R  P  L  A  A  F  L  R  Q  L  Q  S  H  A  P  T
 61 CACGGCGGTGGTCGCCCCCTCGCTGCCTTCCTACGGCAGCTCCAATCCCACGCCCCGACA
 41 S  A  Q  L  F  G  F  L  A  L  F  I  S  G  G  I  L  L  F  L
121 TCCGCGCAGCTATTCGGCTTCCTCGCCCTCTTCATCTCCGGCGGAATCCTCCTCTTCCTC
 61 T  G  V  T  V  T  A  S  V  L  G  F  I  A  F  L  P  L  I  I
181 ACTGGAGTCACCGTCACAGCCTCTGTCCTCGGCTTCATCGCTTTTCTTCCCTTGATCATC
 81 I  S  S  P  I  W
241 ATATCAAGCCCGATCTGgtaatattatatatatgcatgtaatacaatgatttgaatccat
301 gaactttcgaaacagaacaaaatacgagtcaataactcgcttgttttgccgagccatgtg
 87                                                       V  P  A
361 ttttgtacatgacacattaaatactaccaatggaaaagatgtactatgatagGGTCCCGG
 90   V  V  L  L  G  G  F  L  T  V  A  G  S  I  V  A  T  V  A  A
421 CGGTGGTCCTCCTGGGCGGGTTCTTGACGGTCGCTGGTTCGATCGTGGCGACGGTTGCAG
110   V  S  W  T  Y  R  Y  L  R  G  M  H  P  V  G  S  D  Q  V  D
481 CCGTGTCATGGACGTACCGATATCTACGGGGCATGCACCCGGTCGGGTCCGATCAAGTGG
130   Y  A  R  T  R  I  Y  D  T  A  A  H  V  K  D  Y  A  R  E  Y
541 ATTATGCTCGGACTCGGATCTACGACACGGCGGCTCACGTCAAAGACTATGCAAGAGAGT
150   G  G  Y  L  Q  S  K  V  K  D  A  A  P  G  A  *
601 ACGGTGGTTACCTCCAAAGCAAGGTCAAGGATGCGGCTCCTGGTGCCTGA
```

Figure S3.17 The gene model of *GgOLE2*. The coding region is marked with uppercase letters, above which are its deduced amino acids (the oleosin domain is shown in red). The start and stop codons are marked with **bold** letters, whereas the conserved 12-residue proline knot is boxed.

```
  1 M   A   D   Q   N   P   T   Q   R   P   L   Y   G   G   S   G   G   P   H   G
  1 ATGGCCGACCAAAACCCAACCCAGAGACCCCTTTACGGCGGTTCCGGTGGTCCTCACGGC
 21 G   G   R   P   I   S   A   L   L   R   L   L   Q   S   H   G   P   T   S   A
 61 GGTGGCCGCCCCATCTCCGCCCTTCTCCGTCTTCTCCAATCCCACGGTCCCACCTCCGCT
 41 Q   L   F   G   F   L   A   L   F   I   A   G   G   I   L   L   L   L   T   G
121 CAGCTATTCGGCTTCCTCGCACTTTTCATCGCCGGCGGAATCCTCCTTTTGCTCACCGGC
 61 V   T   V   T   A   S   V   L   G   L   I   A   F   L   P   L   I   I   I   S
181 GTCACTGTCACCGCCTCAGTCCTCGGTTTAATCGCCTTTCTTCCTCTCATCATCATCTCA
 81 S   P   I   W
241 AGCCCCATCTGgtaattaattattctttatatggttttgttattataacgcgaaaattac
301 catgcatttcctctaagaatgtattatgcattattcctcgaaataaaaaaaaatatttt
361 ataaaggttttaattgaaatttaaagaaaataaaattgtaaatggacattgatgttagaa
421 ttagtaaaaaaaaagagagaattatatattttgtgaattgttgaaaaaatgaattgtatt
481 ttttaattgaagactacatctattaataaaagaaaatataaaagaaaataaagatacaag
541 aaaaagaaaattaagatataagaaatataagaaaaaaatcttaattgaagattacatcta
601 tttatacaagagggattcctcttgagaatacaaaaaaaatgtaaaagaaaataaaaatac
661 aataaaaaaattatagagaaaaagaagaaagaaaagattaaaaagaaaataatattataa
 85                                                 V   P   M   V
721 caaaattatgttttaataattttaacagtggttgaactctttgacagGGTCCCCATGGTT
 89 V   L   L   C   G   F   L   T   V   A   G   S   T   V   G   T   V   A   V   V
781 GTACTTTTATGTGGGTTCTTGACGGTGGCGGGATCGACGGTGGGTACGGTGGCAGTCGTA
109 S   W   T   Y   R   Y   L   R   G   M   H   P   V   G   S   D   H   V   D   Y
841 TCATGGACGTATCGGTACTTACGTGGCATGCATCCGGTCGGATCGGATCATGTTGATTAT
129 A   R   S   R   F   Y   D   T   A   V   H   V   K   D   Y   A   R   E   Y   G
901 GCCCGGAGTCGGTTCTACGACACGGCGGTTCACGTCAAAGATTATGCCAGAGAGTACGGT
149 G   Y   L   Q   S   K   V   K   D   A   A   P   S   A   *
961 GGTTACCTCCAAAGCAAGGTTAAAGACGCGGCTCCTAGCGCCTGA
```

Figure S3.18 The gene model of *GgOLE4*. The coding region is marked with uppercase letters, above which are its deduced amino acids (the oleosin domain is shown in red). The start and stop codons are marked with **bold** letters, whereas the conserved 12-residue proline knot is boxed.

```
  1 M  A  D  V  Q  A  R  T  H  E  M  S  R  E  H  P  K  S  R  Q
  1 ATGGCCGACGTGCAAGCTCGAACACACGAGATGAGCAGGGAGCATCCCAAGTCCAGGCAG
 21 M  V  K  A  A  T  A  V  T  A  G  G  S  L  L  V  L  S  G  L
 61 ATGGTGAAGGCGGCGACGGCGGTTACGGCCGGTGGATCCCTGCTGGTGCTCTCCGGCCTA
 41 T  L  A  G  T  V  I  A  L  T  V  A  T  P  L  L  V  I  F  S
121 ACACTCGCCGGAACAGTGATCGCTCTCACGGTGGCGACTCCATTGCTGGTCATCTTCAGC
 61 P  V  L  V  P  A  V  I  T  V  A  L  L  I  T  G  F  L  A  S
181 CCTGTCCTCGTCCCCGCCGTCATCACCGTCGCCCTCCTCATCACTGGTTTCCTCGCTTCC
 81 G  G  F  G  I  A  A  I  T  V  F  S  W  I  Y  R
241 GGAGGCTTTGGAATCGCCGCCATTACTGTCTTCTCCTGGATTTACAGgtatacatttata
301 aataggtagatagatatatagacagacatgtgatatgtgcaagatcaaagagccgacttt
 97                         Y  V  T  G  R  H  P  P  G  A  D  K
361 gttgggatcatatataaacatagGTACGTGACGGGGAGGCACCCACCGGGAGCGGACAAG
109 L  D  S  A  R  M  K  L  A  S  K  A  Q  E  M  K  D  R  A  Q
421 CTGGACAGTGCGAGGATGAAGCTGGCGAGCAAAGCGCAGGAGATGAAAGACAGAGCCCAG
129 Q  F  G  Q  Q  H  T  G  G  G  G  A  H  Q  T  S  *
481 CAGTTCGGACAGCAACACACCGGCGGCGGTGGAGCCCACCAGACCTCGTAG
```

Figure S3.19 The gene model of *GgOLE5*. The coding region is marked with uppercase letters, above which are its deduced amino acids (the oleosin domain is shown in red). The start and stop codons are marked with **bold** letters, whereas the conserved 12-residue proline knot is boxed.

1 M A D R E R D R H M Q V G H P Y E G G Q

1 **ATG**GCGGATCGTGAGCGTGACAGGCATATGCAAGTTGGTCATCCTTATGAAGGAGGTCAA

21 G I K S L L P D R G P S T T Q

61 GGCATCAAGAGTCTCCTCCCTGACCGAGGCCCCTCTACCACACAGgtaacctaatgctta

121 atagtataccggaacatatatcctcatatatgaatatatagatacaaaattcattgttat

181 gaccgttggatttcactatttatcttatatgttctttgatggagatactagtttaaataa

241 ttatacacgtacatatatataactcatatatgaatatatagatacgaaattcattgttat

301 gaccgttggatttcactatttatcttatatgttctttgatggagatactagtttaaataa

36 V L A L L A G

361 ttatacacatacatacatatatatatatatatatacacagGTCTTGGCCCTGTTGGCCGG

43 I P V G G T L L A L A G L A L A G S V I

421 GATCCCGGTTGGTGGTACGTTGCTCGCCCTAGCCGGTCTGGCTCTGGCAGGTTCGGTGAT

63 G L M I A T P L F F I F S P V I V P A A

481 TGGTCTGATGATCGCCACGCCGCTCTTCTTCATCTTCAGCCCCGTGATCGTCCCGGCGGC

83 I T I G L A V A G F L S S G L F G L T G

541 GATCACAATCGGGCTCGCGGTTGCGGGCTTCTTGTCTTCGGGCCTGTTTGGGCTTACGGG

103 L S S I S W V M N Y L R L A R R S V P D

601 CTTGAGCTCAATCTCTTGGGTCATGAACTACCTCCGCTTGGCAAGACGGTCCGTGCCCGA

123 Q V D Y A K R R M A D V V G Y A G Q K T

661 CCAGGTGGACTACGCCAAGCGTAGGATGGCCGATGTGGTGGGCTATGCGGGCCAGAAGAC

143 K D M G Q T V Q S K A H E V S R T *

721 GAAGGACATGGGCCAGACCGTGCAAAGCAAGGCCCATGAAGTCTCCAGGACC**TGA**

Figure S3.20 The gene model of *GgOLE6*. The coding region is marked with uppercase letters, above which are its deduced amino acids (the oleosin domain is shown in red). The start and stop codons are marked with **bold** letters, whereas the conserved 12-residue proline knot is boxed.

```
  1 M  A  D  V  R  T  Q  P  H  Q  L  Q  V  H  P  Q  R  H  H  E
  1 ATGGCGGACGTACGTACACAGCCTCACCAACTGCAAGTTCACCCGCAACGTCATCATGAA
 21 G  G  V  K  T  L  L  P  Q  R  G  P  S  A  S  Q
 61 GGCGGCGTCAAAACCCTACTTCCTCAACGTGGCCCTTCTGCTTCTCAGgttaattctttc
121 ttcttcacactcttatatacatacattaggaggtataaacatatatatatatagagagag
 37                              V  L  A  V  V  T  G  I  P  V  G  G  T
181 agagataatgggtttgcatgcagGTTCTGGCAGTTGTGACGGGAATACCAGTGGGGGAA
 50   L  L  T  L  A  G  L  T  L  A  G  S  V  I  G  L  M  V  A  F
241 CACTGTTGACATTGGCCGGTTTAACGCTAGCCGGTTCGGTTATCGGGCTGATGGTGGCTT
 70   P  L  F  V  I  F  S  P  I  I  V  P  A  A  I  T  I  G  L  A
301 TTCCACTGTTTGTGATCTTCAGTCCGATAATTGTACCGGCGGCCATAACCATCGGTTTGG
 90   V  T  G  F  L  A  S  G  A  I  G  L  T  G  L  S  S  M  S  W
361 CAGTAACCGGGTTTCTGGCATCAGGAGCAATCGGGCTAACGGGGTTATCATCGATGTCAT
110   V  L  N  Y  L  S  R  A  T  E  A  V  P  E  Q  L  D  Y  A  K
421 GGGTACTGAACTATCTGAGCCGAGCAACGGAGGCTGTACCAGAGCAACTGGACTATGCGA
130   R  R  M  A  G  M  A  G  Y  V  G  S  K  T  K  D  V  G  Q  S
481 AGCGGCGCATGGCCGGCATGGCTGGATATGTTGGTTCCAAGACTAAAGATGTTGGTCAAT
150   I  E  S  K  A  H  E  V  Q  I  S  T  *
541 CAATTGAGAGCAAAGCCCATGAAGTGCAGATCTCCACATGA
```

Figure S3.21 The gene model of *GgOLE7*. The coding region is marked with uppercase letters, above which are its deduced amino acids (the oleosin domain is shown in red). The start and stop codons are marked with **bold** letters, whereas the conserved 12-residue proline knot is boxed.

```
   1 M  E  D  T  D  R  D  I  T  I  S  G  E  S  H  W  E  I  T  T
   1 ATGGAGGATACAGATAGAGACATAACCATCTCCGGCGAAAGCCACTGGGAAATCACCACT
  21 A  S  A  V  G  T  T  V  A  A  L  A  M  S  G  P  L  L  A  L
  61 GCATCTGCAGTGGGGACTACCGTGGCAGCCTTGGCCATGAGCGGCCCACTTTTGGCTCTG
  41 M  S  F  S  L  L  A  T  M  T  L  F  L  I  A  S  P  V  L  L
 121 ATGAGCTTCAGCTTATTAGCGACTATGACTCTGTTTCTGATAGCTTCGCCGGTACTACTG
  61 I  F  S  P  V  L  L  G  A  L  G  V  L  T  T  A  M  V  G  F
 181 ATATTCAGTCCGGTGTTGTTGGGAGCCTTGGGGGTTCTGACAACGGCTATGGTAGGGTTC
  81 G  V  A  G  A  M  W  V  T  G  L  T  A  L
 241 GGGGTAGCAGGAGCCATGTGGGTAACAGGGCTGACGGCGTTGgtatgatggtcccagatg
 301 ttgtattcaagtagaatctcaataacagatgtatttggtggtaaaagtctacccttttca
 361 ttccataactgaattaaatagcagaatacaaagcttggagtatcaactccttaaaacgtg
 421 gaagtggaaatagtggaagagatgggtgaccaattctctccttatggtgcacttgaaaag
 481 cagagaaaaacaaggaataaaaatagcacttctcgactcaatcatactttcggttcggca
 541 tccttatccggtttgatcttctcggttcaccgacttgttctggttcgatttcaccggcat
 601 tctcttcatgctccgcgtcgctcggttccgctcccgtaaccgcatcattaccgccatccc
 661 gaagaacaatcttgtcctcaagattcagactgggaaagctctgtcgtagccgatcaactt
 721 cttcccaagttgcgtcaacaactggcaagtcttcccattgaacaagggcttctcgtatga
 781 actcgcgcccgtgtgttgggttcgaaaacgcagcacatgcagaggcttaagacgcaacac
 841 tccagtagagcgaacagaggcaatgtactgatgttggtgtgtcgggccgacatgtcgttt
 901 aagaagagacacatgaaaaacattgtgaactctcgatcctctcgtagtcagtaagcgata
 961 tgcaatgtgtccaatgcgagcctcaatccgaaatggcccatagtaacgagtggaaagctt
1021 atgggtgatcgccaacaccgtatgttgtcgataccaagaagcagataaacccattcgcca
1081 actcgaaatgagacatcctgcgtttggatcgaccaccgcttcatccgattaactgcagca
1141 gacaaattctcgctttaactcccgtagcactttctctcgctccataagttgttcgcctag
1201 ctcatggactggtgttgattggccttcgtatcggacaagagctggtggatcgcgaccata
1261 catagctttaaaaggggtcattcctgccgatgcgtgatacgtcgtattataccaatattc
1321 ggcccaaggaatgaagaaactccactgttttggatggcgatgaacaaaacagcgtaaata
1381 ttgctcgatacagcggttaacgacctcggtttgcccgtccgtttgtggatgataagacga
1441 cgacatcttcggatctgtttctgataaacgccacgcttctttccaaaaattactaatgaa
1501 tattggatcccgatcacttacaatagacttgggaatgccatgaagcttcacaatcgaatc
1561 caggaacttggcggcaattcctccggccgtataaggatgtgcggcggtaccaaatgagcc
1621 gctttgacaatcggtccaccacaaccattatcgacgtgttattgtcttaacgaggcaccg
1681 tcaatgaaatccattgatagatcctcccatagccgatttggaacagcaggggttgtaata
1741 ggccttttggcgccatattttcggactttacacgttgacacacatcacattgtgcaacat
1801 agtcgccgatcgatttgtgcatttgtggccaataatactcccgtgataaacgctttaggg
1861 ttcaagcgtgcacagtgaccgccaagtggtgaatcgtggtaatcgtgaagaagggactgg
1921 acgaaagaggagttcggtggaattaccaaacggttacggtaatggaccggcatcgtgacg
1981 cttaatatacggtactcggtgtttcgtatcggcaccgcttgccaattttcacgagatacg
```

2041 gatcaatcatccgctctttgtcgaagatcacgcaaaaaatctgtttgttatctcaaacgg
2101 cattgagacaaggactcccaacgcgacgagagagagcatcaccgttctgtattagccgtc
2161 atgggcttgtaagtgatctcgtaatcataaccgacgagttttcccatccatttttgttgt
2221 tcggagtgaggatcgttgttcaagcagataacggagactcctctcagtctgtttggatag
2281 tgaatcgtcggccaagaaggtaaggacgccaagttcgaaccgcgatcacaatggccaaga
2341 tctcacgcgcataagttgactaactcgctttgcaacgcctaaagagcggctcatgtatgc
2401 aattggcttcccattcgagtaagaatcgccctataccatctccggatgcatcggtttgta
2461 taacgaaaggaatggtaaaatccggaagagctaatgttggtgtcgtagccattctcgctt
2521 tcagtgcaacaaatgcttcttggccatggtgaccaagcaaacttccctttcttgaggagg
2581 tcagtaagtggcacggctatcactccgtagttctcaacgaatttccgataatatcctgtt
2641 aacccgagaaacccacgaagctcggttatggtagtcggtgttggcgagtcgagcatcgct
2701 gaaatcttgttgttatccaccgaaactccaacaccggatataacatgacctaggtagtct
2761 acttttgtttgcccaaaagcacacttcttctgcttaacgaataggttctgcgctttcaaa
2821 atttcaaagacgcgttgaacatggtcgatgtgttcttcccacgaacgattgtagatcaaa
2881 atatcatcaaagaatacgagaacaaatcggcgtagataatcccgaaaaatcgaattcatc
2941 aaagcttggaacgttgacggtgcgttgcataaaccaaagggcatgaccaaatattcataa
3001 tgaccattgtgagttcgaaaagccgttttgtggacatcggagggatgaactctaacttgg
3061 tgatagccatttgtaaggtcaagtttggtaaaaatggtggccccgtgaagttcatccagc
3121 atatcgtcgacggttggaatcggaaatcgatctttactgatgatgacatttaaagctcga
3181 taatcggtgcgaaaccgccaagacccgtctttcttcttcacgaaagcaagactgagagcg
3241 agaatgggcttgaactctactgaataattccagcctcccaaagatctcttttacttgacg
3301 ttcgatctcttctttttggaaatgggcgatcggtaaggacgcacgttgactcggtcgcat
3361 ccttcctttagcgtaatccgatgctcaatcagccggtgtggtggcaattgcgttgggaaa
3421 tcgaaaattgatgaatattgttgcagcagaggatgaagttgctctggaacagaaggagca
3481 acgccgccttcaggatgataggtaacaagaaagcgaggccgagctttgtttagcctcctt
3541 aatgaattccttaggttggggtatgcgaatgggtttgtcttgcacccgtgaataataatc
3601 cttttaccgcacaaagtaaattccaaggtttgggccttccaatcacataatgtaggccca
3661 agttgagttagccattgaatacccaaaacaagatcccgcccttttagaggtaaaggatag
3721 aaatcgatggagaaactcacgccgccgatggaggtgaggatttgccgatatgctcctgtg
3781 caatggattggatgtccgtccgccactctcacctgaaacggcttcgttggtgtgagttgc
3841 atattgagccgcttagccactttgtctgtgataaaattatgggttgagccactatcgatc
3901 aatgcgacgagcttggtgcgatcgatttcagcctgggcccgaagggttttcggagagccc
3961 catccagtaagggcgtgaacggtgatttctggttcttcggctccggaccggtttcgcgga
4021 atcccgtcgtcatccgaatcatcgattccttcaatcaataacggtgagatttcggcatcg
4081 atggccaaggtgtaacgcttcatcacaaccaaacagacccaagctctttttcgcttcatt
4141 tcatcccaactaagcctcttgggggttacatttgttgtagctggtggtagagaagacaga
4201 ccactcgtattgggtttggacgttgtgcttcgttgaccatgatgcacaaaagaagtggtt
4261 tttctttggcgttatgaatcggtcatctctcatgcgtgctaattcaatggtggtcttcaa
4321 ggttttcggttgaaacatacgaatgtcagtcagagatgttctctttaagccccgatgtaa
4381 gttccaataagtgcttttgagtccaccggataccttgttcgtagtctctcaaattcgcgt
4441 tggtagtcacgcaggttccggttgccttattttcgatagagcttcacaaaatcttctgcc
4501 gccgatggcccgaaccgagcccacaactcttcttcaagacttcctgtaattagaacgttg
4561 tcttcggtgagtgccttcgttgtggctcgccaccactcatttgcctcgtcttctaagtgg
4621 tacgaggcaaagcttacgctaccgtcatgtggaagatcctggtaagtaaagtattgtttc

4681 gctttgcttagccatgcggtcggatcgccttcgctgaattttgggaataccaccttgacg
4741 tgtcgttgattcgcaggtgtcgcaaacggattagtcggttccttttgaacggaggctgag
4801 ggttggtcgtcgttgtttcgtcggtgacctcgtggtgcactagagcctcctcacgcagct
4861 cgagacgacctcggatagcatttggcgaaaggattcctccatgttgcgtattccatcgtt
4921 gattttcattatctcgtcttgaaccatgccgagaccgtgttcaagatcagataatcgctc
4981 tttgttgcttgccatgactctaatgtaaggcttcaagtcacaacaattaggaaacagaag
5041 aacacggctctgataccaattgatggtcccggatgttgtattcaagtagaatctcaataa
5101 cagatgtatttggtggtaaaagtctaccctttttcattccataactgaattaaatagcaga
5161 atacaaagcttggagtatcaactccttaaaacgtggaagtggaaatagtggaagagatgg
5221 gtgaccaattctctccttatggtgcacttgaaaagcagagaaaaacaaggaataaaaata
5281 gcacttctcgactcaatcatactttcggttcggcatccttatccggtttgatcttctcgg
5341 ttcaccgacttgttctggttcgatttcaccggcattctcttcatgctccgcgtcgctcgg
95 V Y V G R Q I G V G G G
5401 ttccgctcctgtaaccgcatcatgGTATATGTCGGACGACAGATTGGAGTGGGAGGAGGT
107 V V E R M V E S A G V R V K D I E N D R
5461 GTGGTGGAGCGGATGGTGGAGTCGGCGGGTGTGAGGGTAAAGGATATAGAGAATGACCGG
127 R G Y L W D K S E D D F S *
5521 AGAGGTTACTTGTGGGACAAGTCAGAGGATGATTTTTCT**TAA**

Figure S3.22 The gene model of *ThOLE1*. The coding region is marked with uppercase letters, above which are its deduced amino acids (the oleosin domain is shown in red). The start and stop codons are marked with **bold** letters, whereas the conserved 12-residue proline knot is boxed.

```
  1 M  A  D  R  R  P  I  Y  G  G  V  G  G  G  S  H  P  I  A  D
  1 ATGGCTGACCGGAGACCCATCTACGGTGGCGTCGGCGGCGGCAGTCACCCCATCGCCGAT
 21 L  L  R  Q  L  Q  T  H  V  P  T  S  G  Q  L  F  G  F  L  A
 61 CTCCTCCGGCAACTGCAAACCCACGTCCCAACCTCGGGCCAGCTATTCGGCTTCCTCGCA
 41 L  F  I  S  G  G  I  L  L  F  L  T  G  V  T  V  T  A  S  V
121 CTCTTCATCTCCGGCGGAATCCTCCTCTTCCTCACCGGTGTCACCGTCACCGCCTCCGTC
 61 L  G  F  I  A  F  L  P  L  I  I  L  S  S  P  I  W
181 CTCGGCTTCATCGCTTTTCTTCCCCTCATCATCCTCTCAAGCCCTATCTGgtaatttatg
241 attctccataacactgaatctccaaaattgtagttttggatccataatctttgagaacag
301 aaatcttgaatctctagagggttttatttttatttatcttcgtctatatatgtgcccta
361 atcttcatctcaaaatccagatgcgttaatcctaaccctagactaacaggaccccccaat
421 cacatactcgtgatgttacaatcaccattttgattattcatcgtttcaaattacacacac
481 acacagtattcttattatctatagatacgatttattatttctagtacgttgactggtcgt
541 gttaatgatgtggcattttatgatagtgattcgtatgtgataataattgacatatcgaaa
 78          V  P  M  V  F  L  L  G  M  F  L  A  V  G  G  S  V
601 tttgacagGGTCCCTATGGTCTTCCTCCTGGGTATGTTCTTGGCGGTCGGGGGATCGGTC
 95 V  A  V  V  A  V  V  T  W  T  Y  R  Y  L  R  G  M  H  P  V
661 GTTGCAGTGGTTGCGGTCGTGACGTGGACGTACAGGTATCTACGTGGCATGCACCCGGTC
115 G  S  G  Q  V  D  Y  A  R  A  R  I  Y  D  T  A  A  H  V  Q
721 GGATCGGGACAGGTGGATTATGCCCGGGCTCGAATTTACGACACGGCGGCTCACGTCCAA
135 V  Y  A  R  D  Y  G  G  Y  L  Q  S  K  V  K  D  A  A  P  G
781 GTTTACGCCAGAGATTACGGCGGTTACCTCCAAAGCAAGGTCAAAGATGCGGCTCCTGGT
155 A  *
841 GCCTGA
```

Figure S3.23 The gene model of *ThOLE2*. The coding region is marked with uppercase letters, above which are its deduced amino acids (the oleosin domain is shown in red). The start and stop codons are marked with **bold** letters, whereas the conserved 12-residue proline knot is boxed.

1 M A D G N P N Q I P I Y G G G G H N A A
1 **ATG**GCCGACGGAAACCCTAACCAGATCCCCATCTACGGCGGCGGCGGCCACAACGCAGCG
21 A L L R Q L R S H G P T S A Q L F G F L
61 GCACTCCTCCGGCAGCTCCGGTCCCACGGCCCGACCTCGGCGCAGTTATTCGGTTTCCTC
41 A F F I S G G I L L F L T G V T V T A S
121 GCATTCTTCATCTCCGGCGGGATCCTCCTCTTCCTCACCGGCGTCACTGTCACCGCCTCC
61 V L G F I A F L P L I I I S S P V W
181 GTCCTCGGTTTCATCGCTTTTCTACCATTGATCATCATCTCAAGCCCGGTCTGgtaatat
241 ttcatgattcaccccacgtattatgcatgcatgcaaaacaatggtctaatttgatttcgt
301 tagggtttgttttcctatattttcttctttgtatttaagataatttgttgataaatacat
361 agtaaatttcatttacgagacacgatcggatgatatttcaataaatctgaatccttgatt
421 cacattgtacatgtgaattgaataaataatgcattacagtttggatcattgaactttgaa
481 catcttttatcaaaaaaaaaaaactttgaacatctttaacaaaaaaaaactttgaacata
541 tggattgaatgaattgaatctgtgatatttttaaaaaaattgttcaaaaactcgaaacca
601 cacatacataaaatcttgcaacacaaaaaaaaaaaatacaaattaacgcatgaaatttcac
79 V P V V V L V G G F L T V A G S I V G
661 agGGTCCCCGTGGTGGTCCTCGTGGGCGGGTTCTTGACCGTGGCGGGATCGATCGTGGGG
98 T A A V V S W T Y R Y L R G M H P V G S
721 ACAGCTGCGGTCGTGTCGTGGACGTACAGGTATCTACGTGGCATGCACCCGGTCGGGTCG
118 D Q V D Y A R T R I Y D T A A H V K D Y
781 GACCAGGTAGATTATGCCCGGACTCGGATCTATGATACGGCGGCTCACGTCAAAGACTAT
138 A R E Y G G Y L Q S R V K D A A P G A *
841 GCAAGAGAGTACGGTGGTTACCTCCAAAGCAGGGTCAAGGATGCGGCTCCTGGTGCC**TGA**

Figure S3.24 The gene model of *ThOLE4*. The coding region is marked with uppercase letters, above which are its deduced amino acids (the oleosin domain is shown in red). The start and stop codons are marked with **bold** letters, whereas the conserved 12-residue proline knot is boxed.

```
  1 M  A  D  V  Q  P  R  P  L  E  M  S  R  E  Q  P  K  S  R  Q
  1 ATGGCCGACGTGCAACCTCGACCGCTGGAGATGAGCAGGGAGCAGCCCAAGTCGAGGCAG
 21 M  V  K  A  A  T  A  V  T  A  G  G  S  L  L  V  L  S  G  L
 61 ATGGTGAAGGCGGCGACGGCTGTGACCGCCGGTGGGTCCTTGCTGGTTCTCTCCGGCCTG
 41 T  L  A  G  T  V  I  A  L  T  V  A  T  P  L  L  V  I  F  S
121 ACGCTCGCCGGAACGGTGATCGCTCTCACGGTGGCGACTCCACTGCTCGTCATCTTCAGC
 61 P  V  L  V  P  A  V  I  T  V  A  L  L  V  T  G  F  L  A  S
181 CCCGTCCTCGTCCCCGCCGTCATCACCGTCGCTCTCCTCGTCACCGGATTCCTAGCCTCC
 81 G  G  F  G  I  A  A  I  T  V  F  S  W  I  Y  R
241 GGCGGCTTCGGCATCGCCGCCATTACCGTCTTCTCCTGGATTTACAGgtatatatatata
301 tacataaaaatacatatatatgtgtgtatggagtggtaatgacgaagggtatttgaagca
 97   Y  V  T  G  R  H  P  P  G  A  D  K  L  D  S  A  R  L  K  L
361 gGTACGTGACGGGGAGGCACCCACCAGGGGCGGACAAGCTGGACAGTGCGAGGCTGAAGC
117   A  S  K  A  Q  E  M  K  D  R  A  Q  Q  F  G  Q  Q  H  T  G
421 TGGCGAGCAAAGCGCAGGAGATGAAAGACAGAGCTCAGCAGTTCGGACAGCAACACACCG
137   G  V  G  A  H  Q  T  S  *
481 GCGGCGTTGGAGCCCACCAGACCTCCTAG
```

Figure S3.25 The gene model of *ThOLE5*. The coding region is marked with uppercase letters, above which are its deduced amino acids (the oleosin domain is shown in red). The start and stop codons are marked with **bold** letters, whereas the conserved 12-residue proline knot is boxed.

1 M A E V Q A R P Q E M S S S W E Q Q P K

1 **ATG**GCAGAGGTGCAAGCTCGACCGCAAGAAATGAGCAGCAGCTGGGAGCAGCAGCCCAAG

21 S R Q M V K V A T A V T A G G S L L V L

61 TCACGGCAGATGGTGAAGGTGGCGACGGCGGTGACGGCCGGCGGGTCACTGCTCGTACTC

41 S G L T L A G T V I A L T V A T P L L V

121 TCCGGCCTGACACTCGCCGGAACGGTGATCGCTCTGACGGTGGCGACGCCTTTGCTCGTG

61 I F S P V L V P A V I T V A L L I T G F

181 ATCTTCAGCCCCGTCCTCGTCCCCGCCGTCATCACCGTCGCCCTCCTCATCACCGGCTTC

81 L S S G G F G I A A I T V F S W I Y R

241 CTCTCCTCCGGCGGATTCGGCATCGCCGCCATTACCGTCTTCTCCTGGATCTACAGgtaa

301 atctgtacatacacatacacaaaatgtatagaaataaccaatgcatgtagcattaaatcc

361 gaacctggaattgggtttaagccgtaatctacgtagggttctgtaacgtcgtgtgattag

421 ataagaatgtgatgaaataaactgggattagttttgtgtaagtattgatgggtgtgtgtg

100 Y V T G K H P

481 gacatatacgtgtatgtatgtatacgtaatacgtatagGTACGTGACGGGGAAGCACCCA

107 P G A D K L D S A R M K L A S K A Q D L

541 CCGGGGGCGGACAAGCTGGACAGTGCAAGGATGAAGCTGGCGAGCAAAGCGCAGGATCTT

127 K D R A Q Q F G Q Q H S A T H Q T S *

601 AAAGACAGAGCTCAGCAGTTCGGACAGCAACACTCTGCCACTCACCAGACTTCT**TAG**

Figure S3.26 The gene model of *ThOLE6*. The coding region is marked with uppercase letters, above which are its deduced amino acids (the oleosin domain is shown in red). The start and stop codons are marked with **bold** letters, whereas the conserved 12-residue proline knot is boxed.

```
  1 M  A  D  R  D  R  E  R  H  M  Q  V  S  H  P  Y  E  G  G  Q
  1 ATGGCGGATCGCGACCGTGAGAGGCACATGCAAGTTAGTCACCCTTACGAAGGCGGTCAG
 21 G  I  K  S  I  L  P  E  R  G  P  S  S  T  Q
 61 GGCATCAAGAGCATCCTTCCTGAACGTGGCCCCTCTAGCACTCAGgtcacataatgcatt
121 tttataaactttttttatcttataattatgttacaatatgtatgtttacaaaaaaaaaagc
181 gtatccatgaaatatctttcttggttataaaccatatatgtagtataatttacattgatt
241 tgatcttccaccgcttaacgcaagtgccatgcacacgcatatgaccagaatgcaagagaa
301 atacgcatctgcatgcatgtcgattgattcgaataaatcagacatttttttcctcgcatat
 36          V  L  A  L  L  T  G  I  P  V  G  G  T  L  L  A  L  A
361 atacgcagGTTTTGGCCCTCTTGACCGGTATCCCGGTAGGAGGCACGCTCCTCGCCCTGG
 54   G  L  A  L  A  G  S  V  I  G  L  M  I  A  T [P  L  F  I  I]
421 CCGGTCTGGCCTTGGCCGGTTCGGTGATCGGCCTGATGATCGCCACCCCACTCTTCATCA
 74  [F  S  P  V  I  V  P] A  A  I  T  I  G  L  A  V  T  G  F  L
481 TTTTCAGCCCGGTCATCGTCCCTGCCGCGATCACGATAGGGCTCGCCGTGACTGGGTTCC
 94   S  S  G  L  F  G  L  T  G  L  S  S  I  S  W  V  M  N  Y  L
541 TGTCGTCGGGCCTGTTCGGGCTGACGGGTCTGAGCTCGATCTCGTGGGTAATGAACTACC
114   R  G  T  R  R  S  M  P  E  Q  M  E  Y  A  K  R  R  M  A  D
601 TGCGTGGCACAAGACGGTCCATGCCCGAGCAGATGGAATATGCCAAGCGTAGGATGGCTG
134   V  V  G  Y  T  G  Q  K  T  K  D  V  G  Q  T  V  Q  S  K  A
661 ACGTGGTCGGATACACTGGTCAGAAGACCAAGGACGTCGGCCAGACCGTTCAGAGCAAGG
154   Q  E  T  S  R  T  *
721 CCCAAGAAACCTCCAGGACCTGA
```

Figure S3.27 The gene model of *ThOLE7*. The coding region is marked with uppercase letters, above which are its deduced amino acids (the oleosin domain is shown in red). The start and stop codons are marked with **bold** letters, whereas the conserved 12-residue proline knot is boxed.

```
  1 M  A  D  V  R  T  Q  P  H  Q  L  Q  V  H  P  Q  R  Q  H  E
  1 ATGGCGGACGTCCGTACACAACCCCATCAACTCCAAGTTCATCCACAACGCCAGCACGAA
 21 G  G  V  K  T  L  L  P  Q  R  G  P  S  A  T  Q
 61 GGCGGCGTGAAAACCCTCCTTCCCCAGCGTGGCCCCTCTGCTACTCAGgtgattaatttg
121 tctccctttggagcccatcaccatcaggcggcatatatatatatatatatatatatatat
181 atatatatacacacacacataacataaacatgtacatgcatatatgtgaatattgtttgc
 37         V  L  A  V  V  T  G  I  P  V  G  G  T  L  M  I  L  A
241 atgcgcagGTGCTGGCGGTGGTGACGGGGATACCGGTGGGGGGGACACTGATGATACTGG
 55  G  L  T  L  A  G  S  V  I  G  L  M  V  A  F [P  L  F  V  I]
301 CGGGTTTGACACTAGCAGGTTCGGTGATAGGGTTGATGGTGGCGTTTCCATTGTTCGTGA
 75 [F  S  P  I  I  V  P] A  A  I  A  V  G  L  A  V  M  G  F  L
361 TATTCAGTCCGATAATAGTGCCGGCGGCCATTGCGGTAGGACTGGCGGTGATGGGATTTC
 95  A  S  G  A  I  G  L  T  G  L  S  S  M  S  W  V  L  N  Y  L
421 TGGCGTCGGGGGCAATAGGGCTGACGGGTTTGTCGTCAATGTCGTGGGTTCTCAACTATC
115  S  R  A  K  E  A  V  P  E  Q  I  D  Y  A  K  R  R  M  A  G
481 TGAGCAGAGCGAAAGAGGCTGTCCCTGAACAGATCGACTATGCCAAGCGCAGGATGGCTG
135  V  A  G  Y  V  G  S  K  T  K  D  V  G  Q  S  I  E  T  K  A
541 GCGTGGCTGGCTACGTCGGTTCCAAGACTAAGGATGTCGGCCAATCTATAGAGACCAAGG
155  H  E  V  Q  I  S  T  *
601 CTCATGAAGTGCAGATTTCTACATGA
```
