# Supplementary figures and images for: Integrative Analysis of Oleosin Genes Provides Insights into Lineage-Specific Family Evolution in Brassicales
Source: Plants (Basel). 2024 Jan 18;13(2):280. doi: 10.3390/plants13020280 (PMC10820149; doi:10.3390/plants13020280)

**Figure S1 Kyte–Doolittle hydrophobicity plots of identified oleosins using ProtScale.**

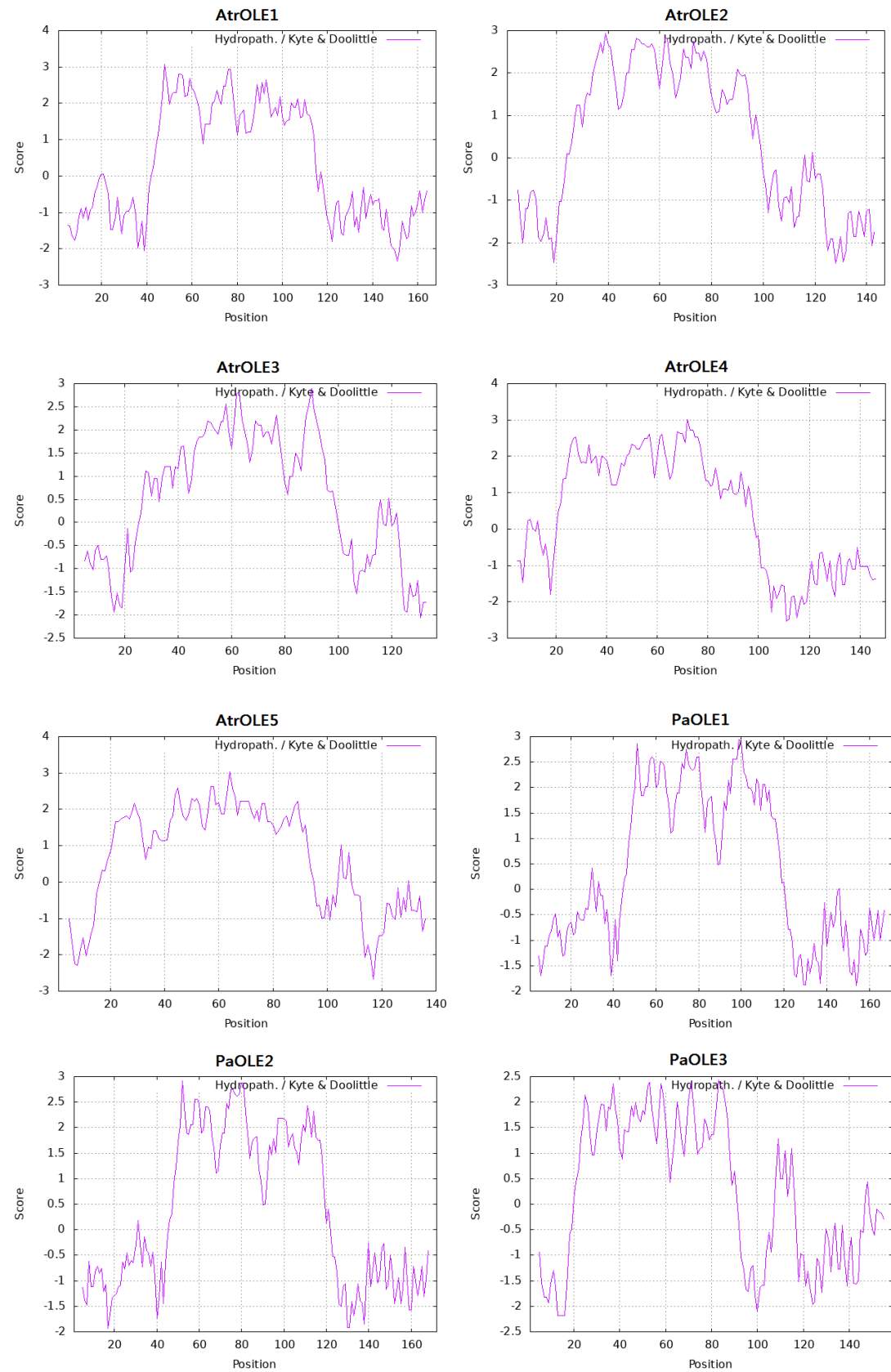

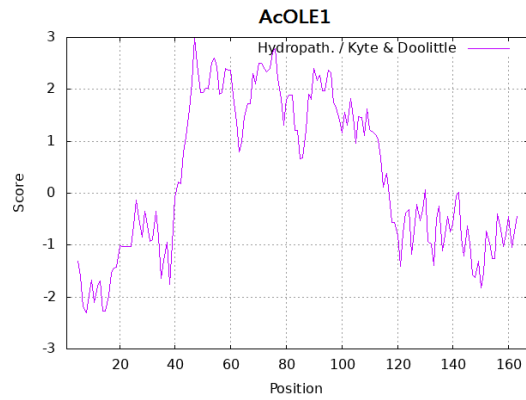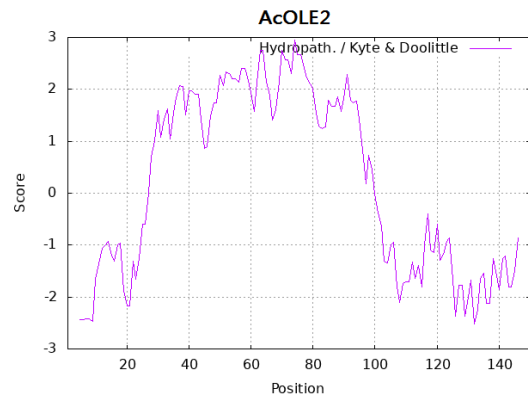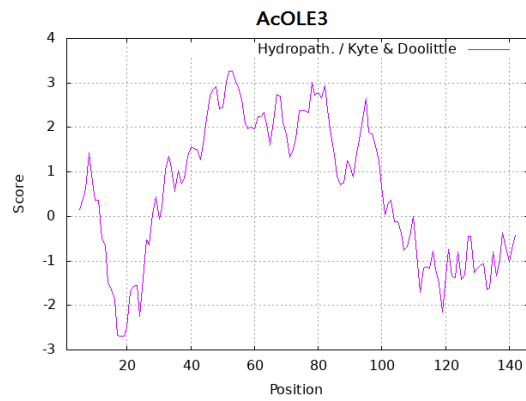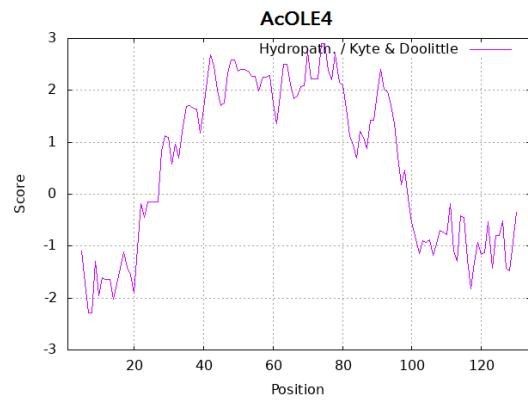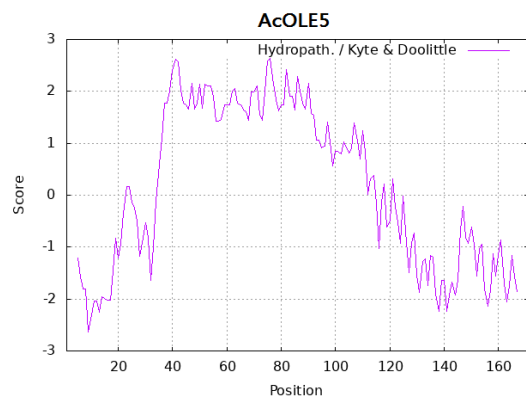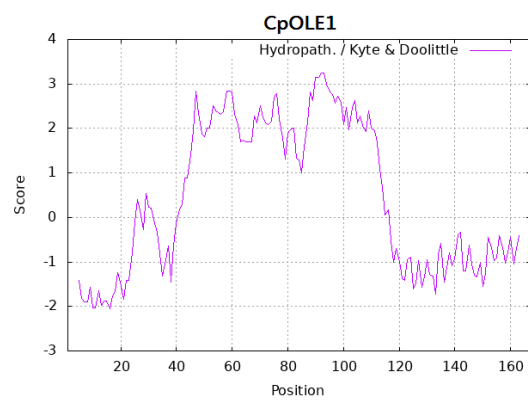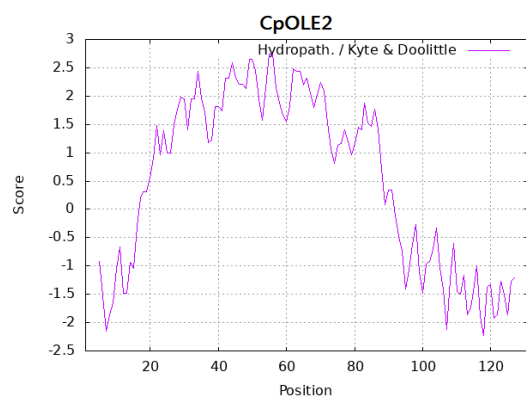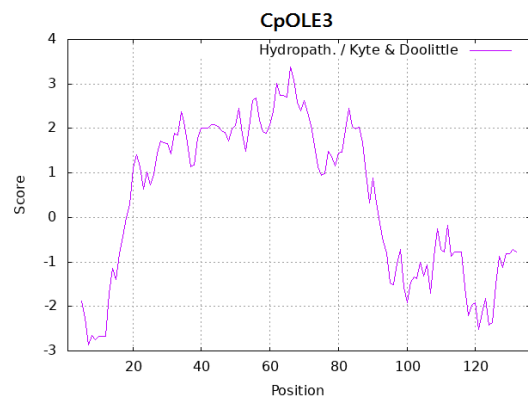

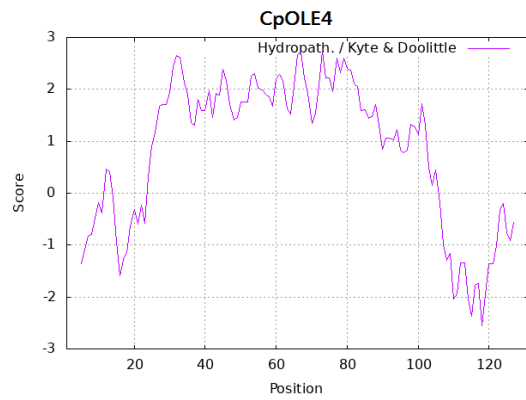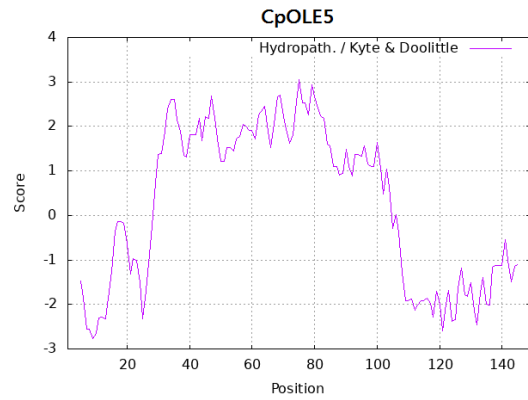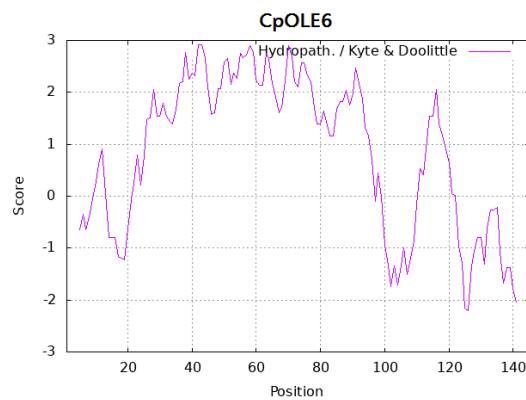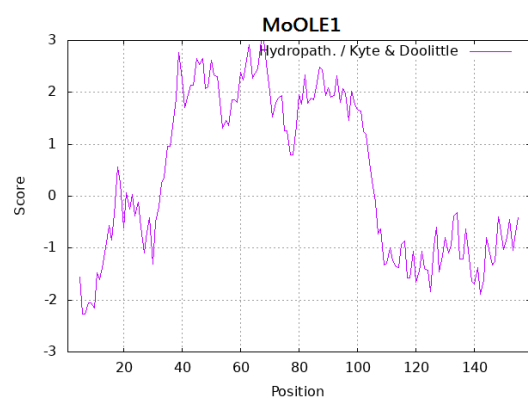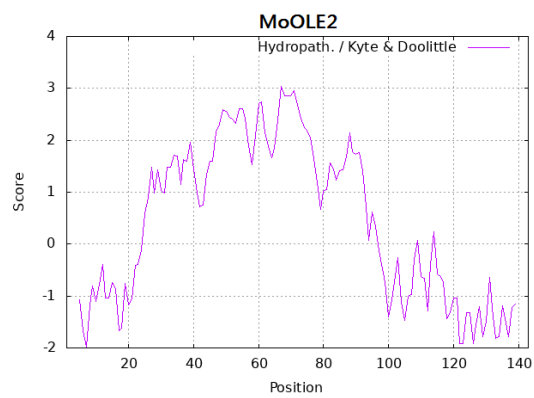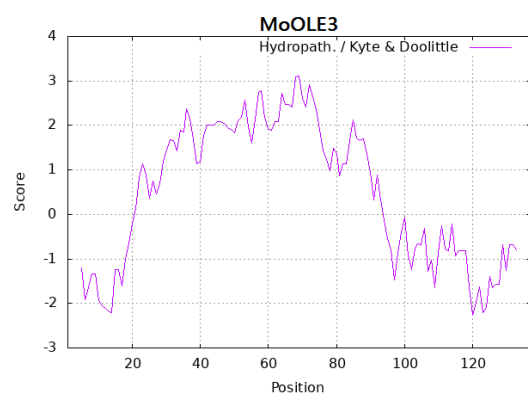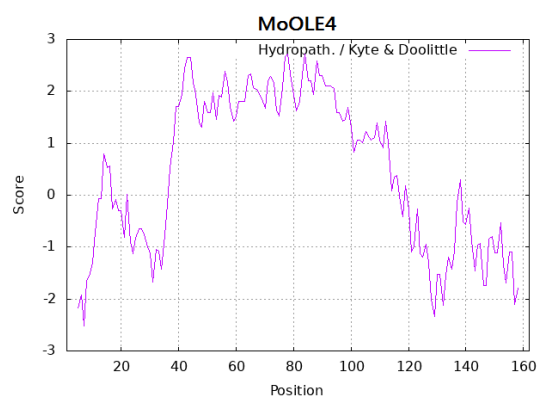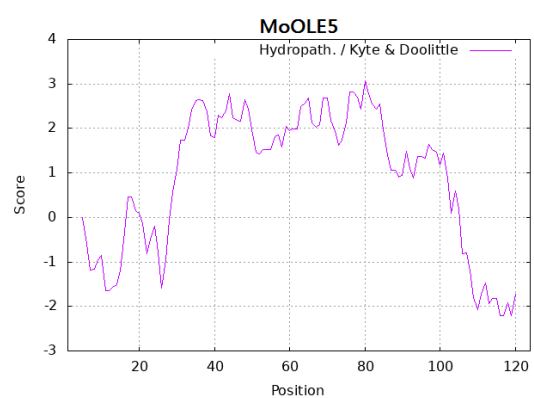

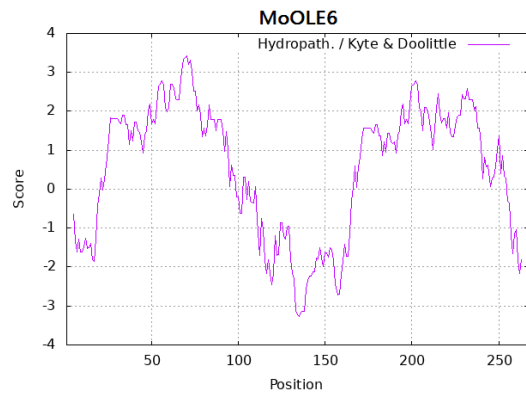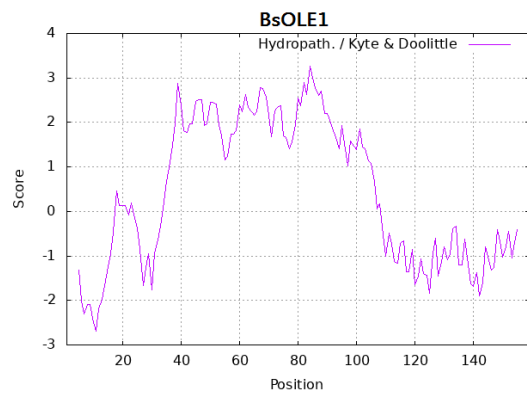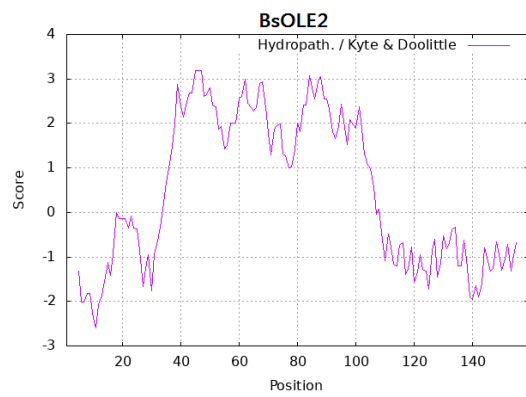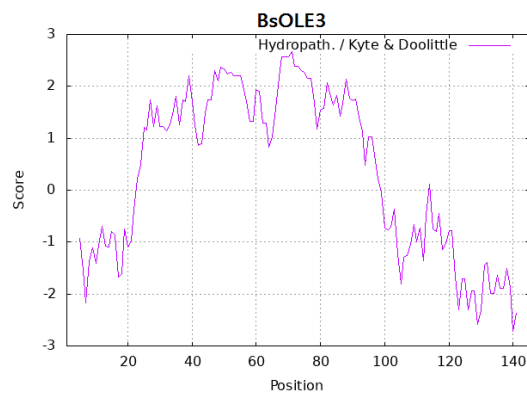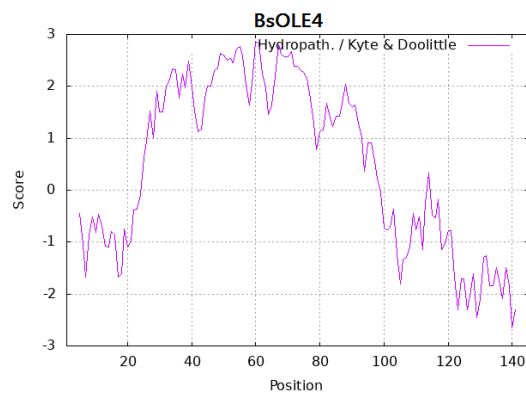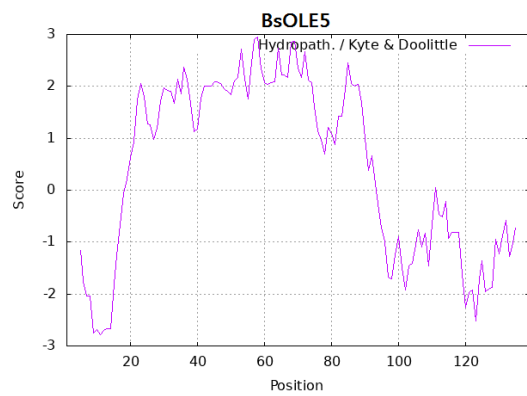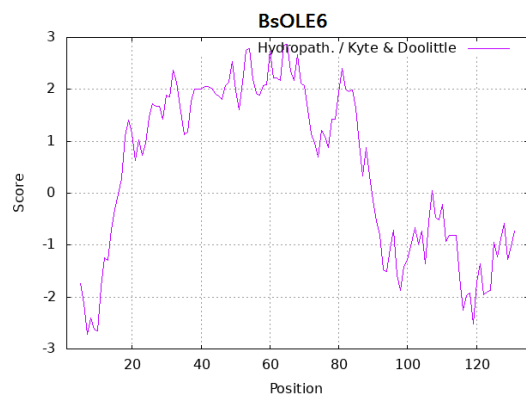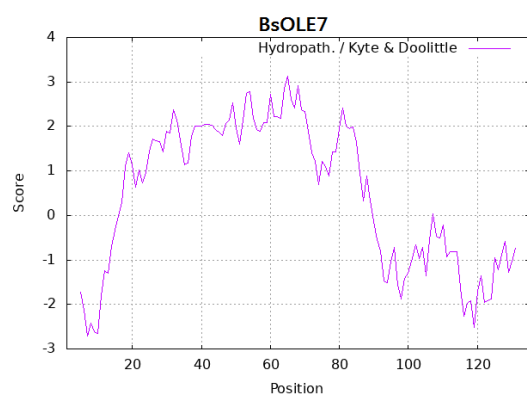

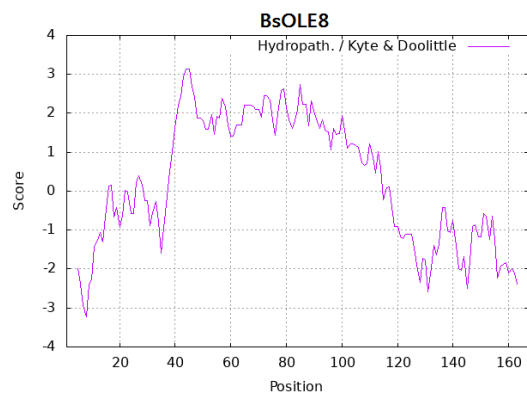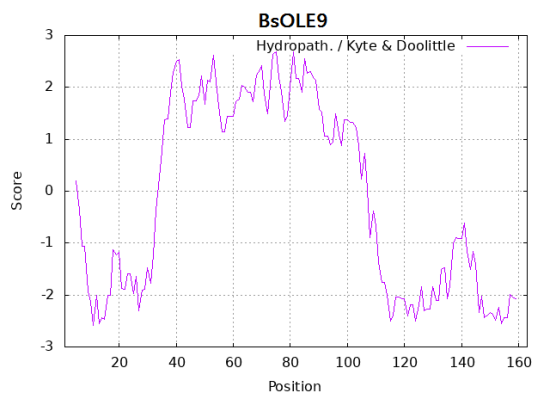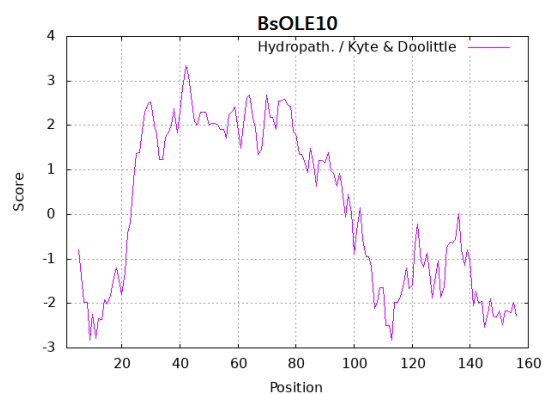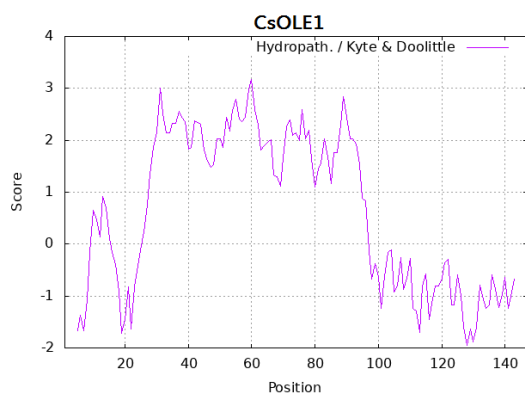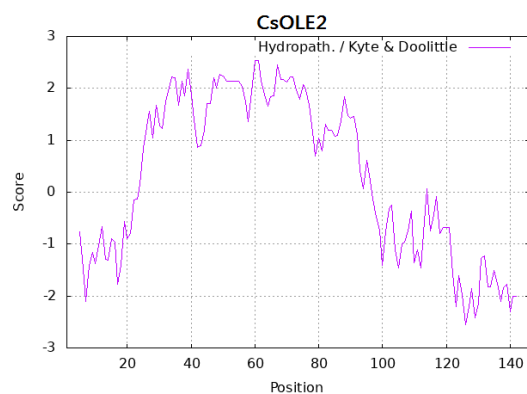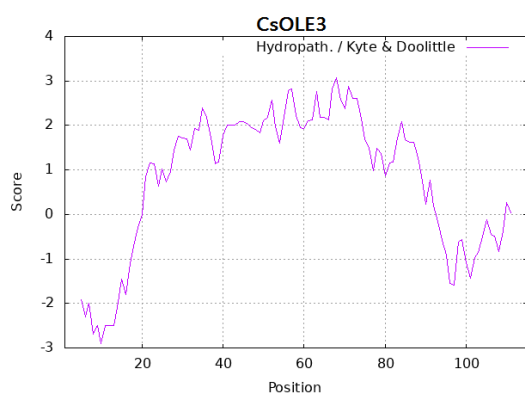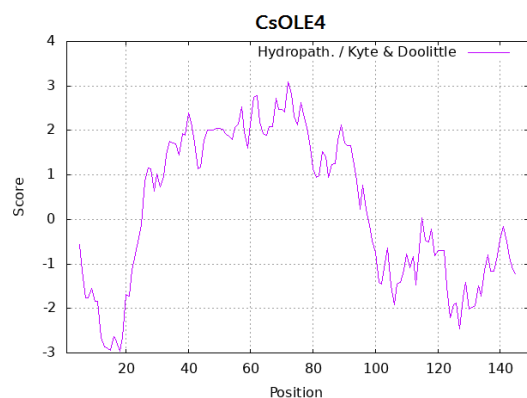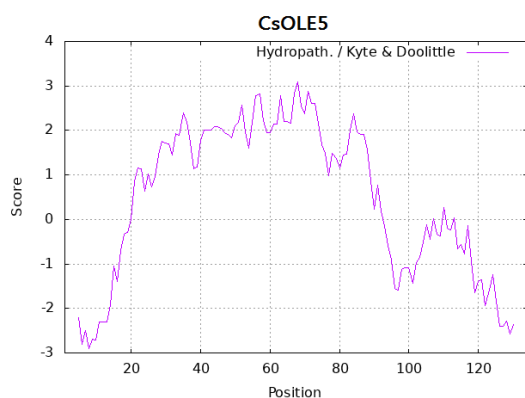

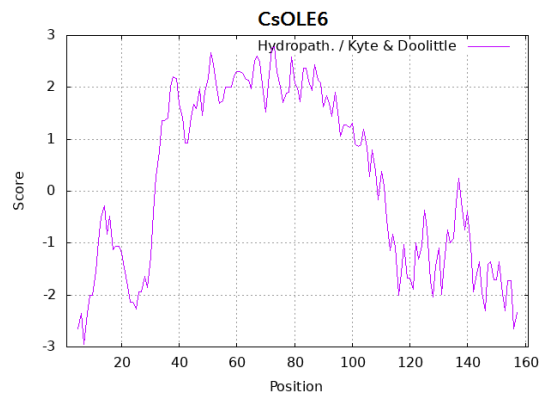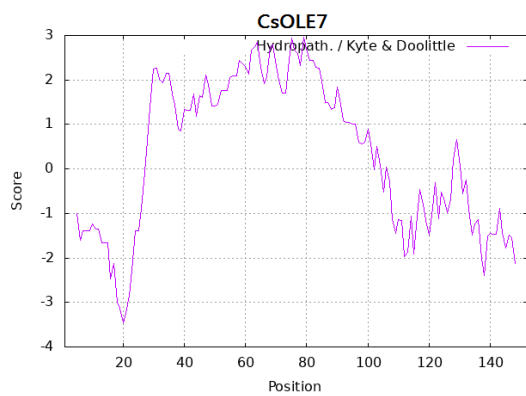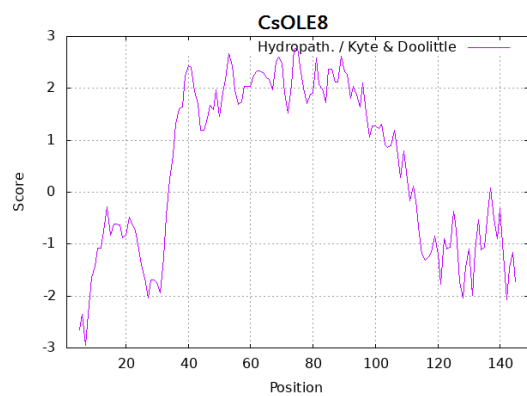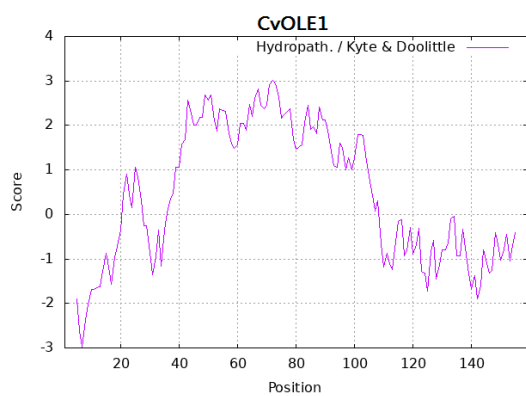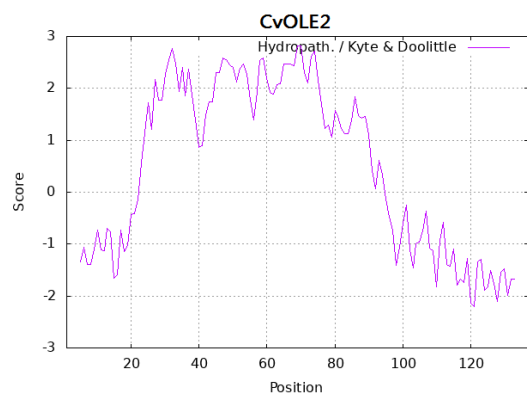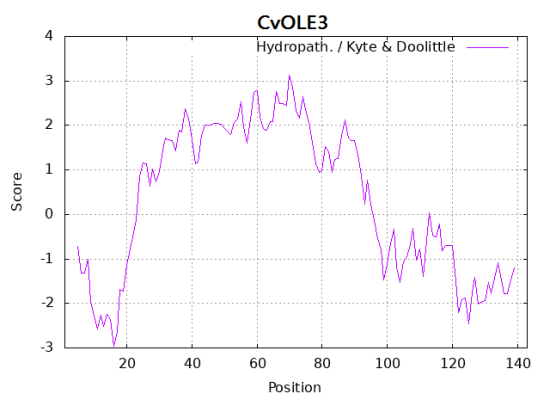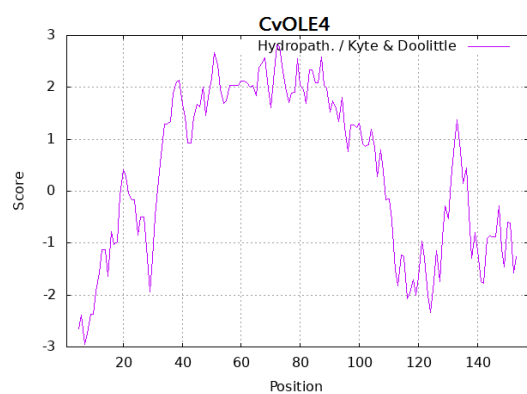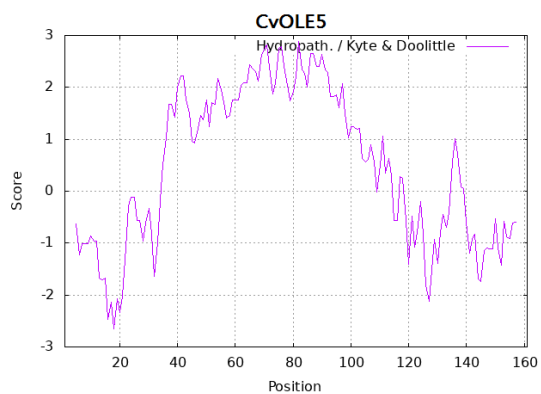

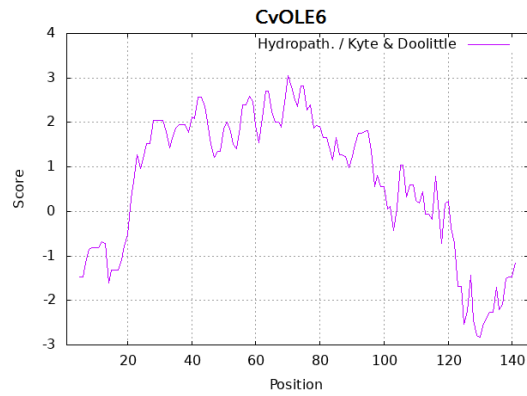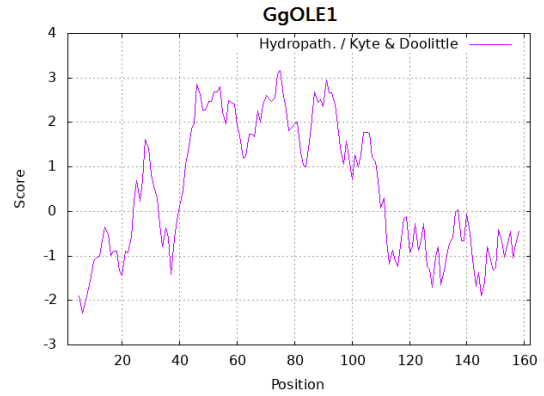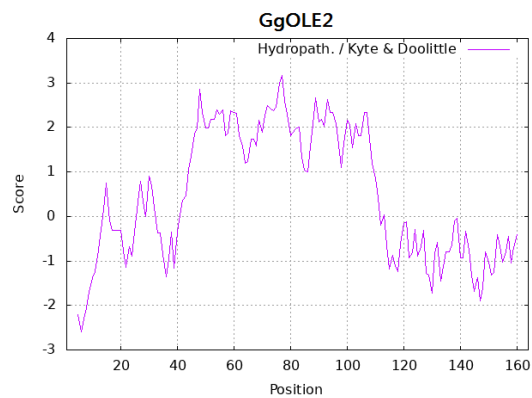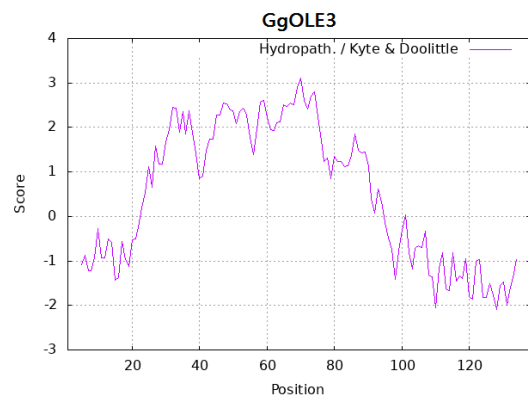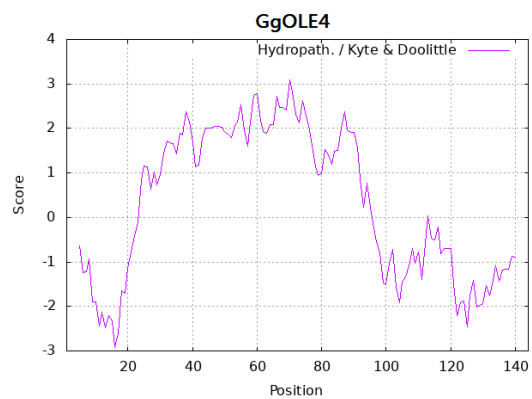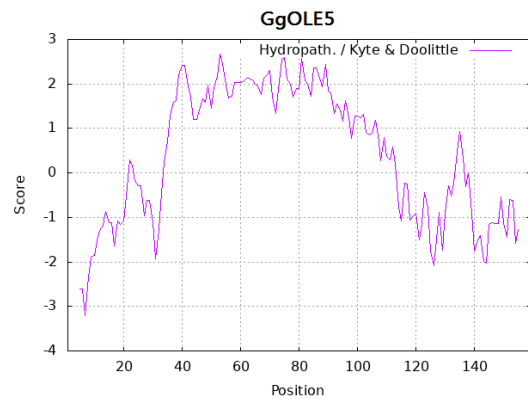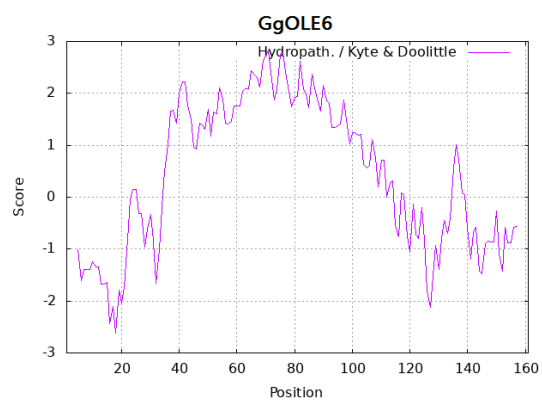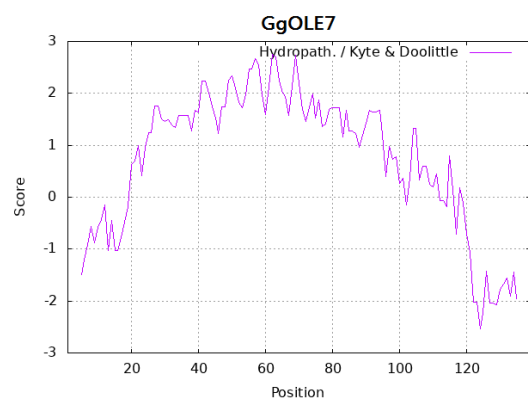

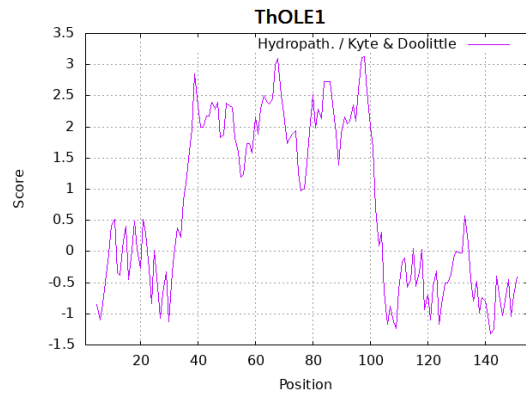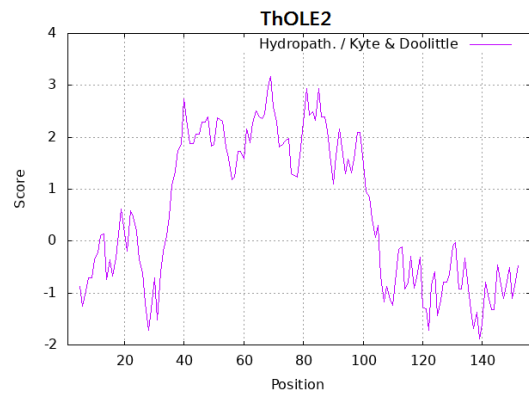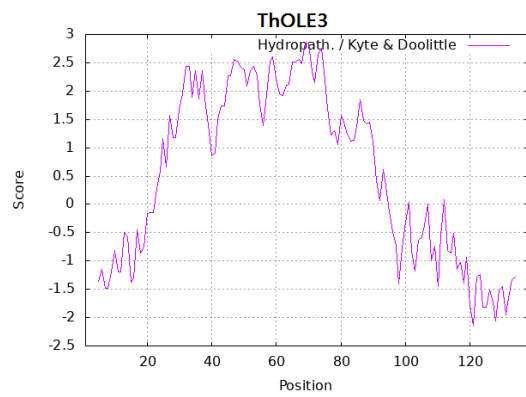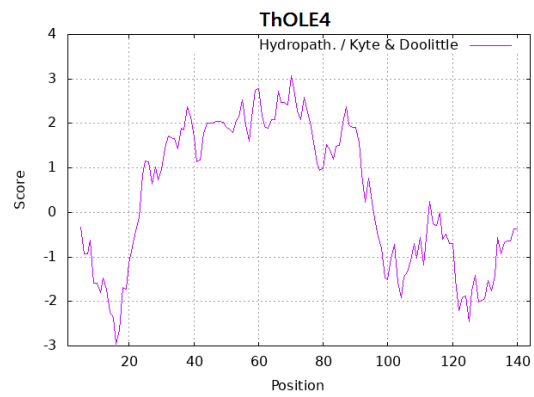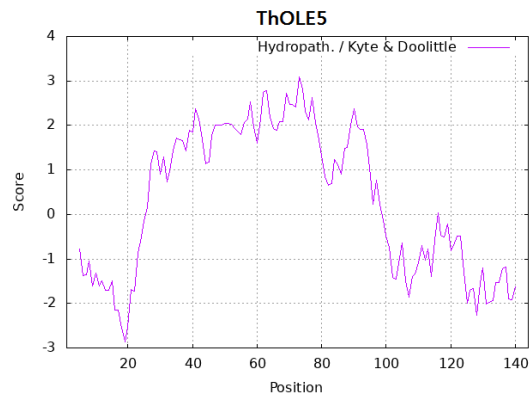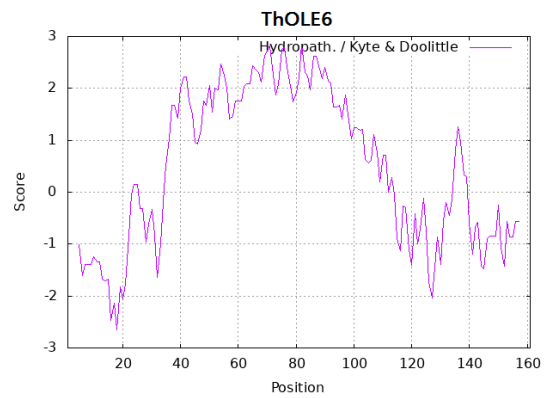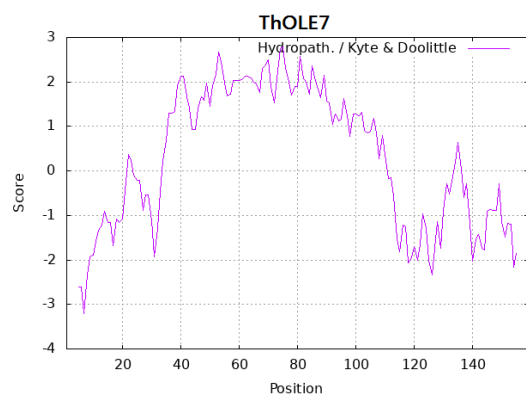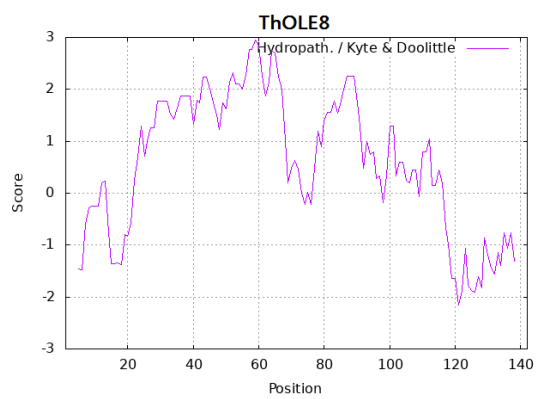

Supplement: Supplementary file 1 [file plants-13-00280-s001.zip › Figure S1.pdf]

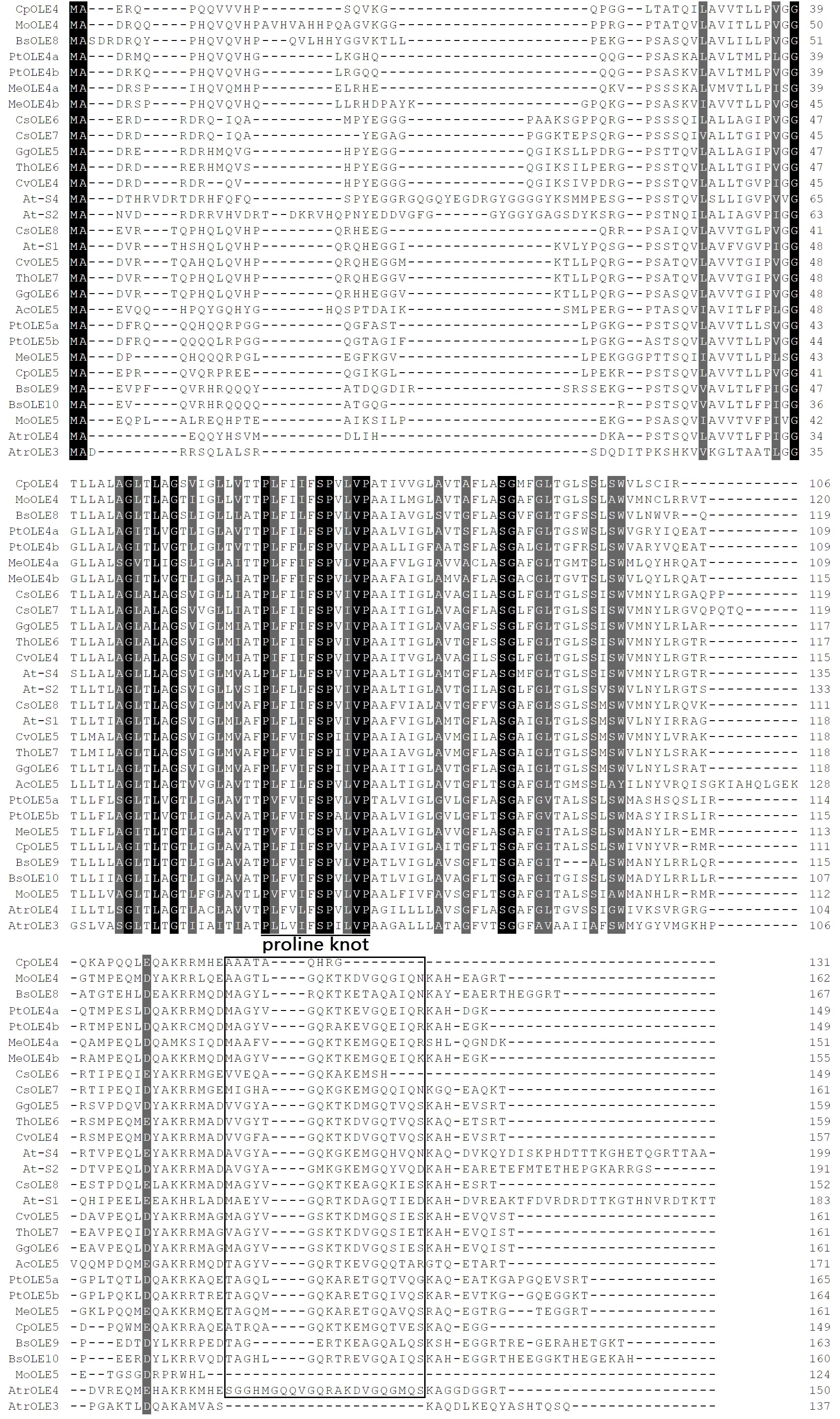

Supplement: Supplementary file 1 [file plants-13-00280-s001.zip › Figure S2.jpg]

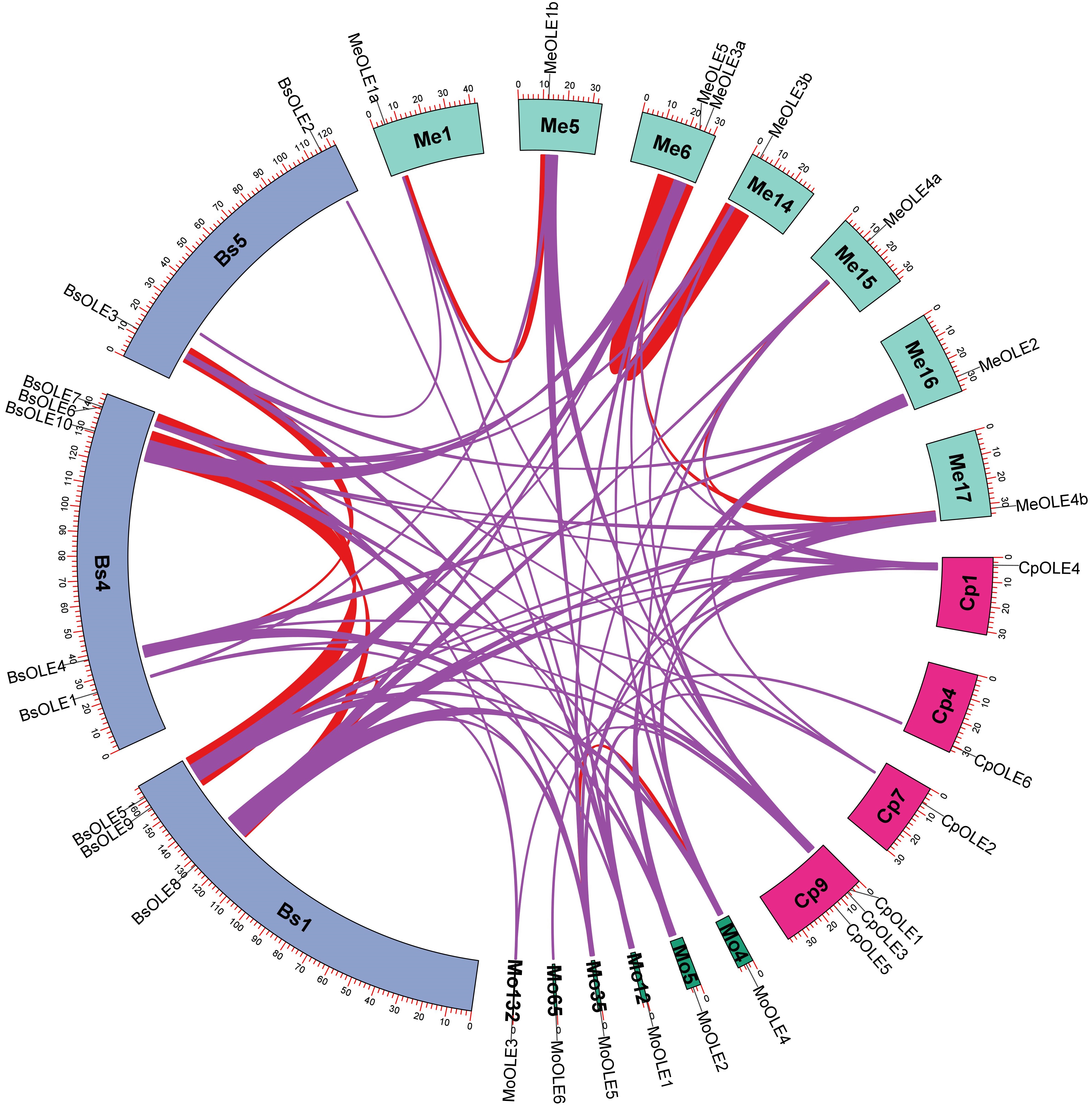

Supplement: Supplementary file 1 [file plants-13-00280-s001.zip › Figure S4.jpg]

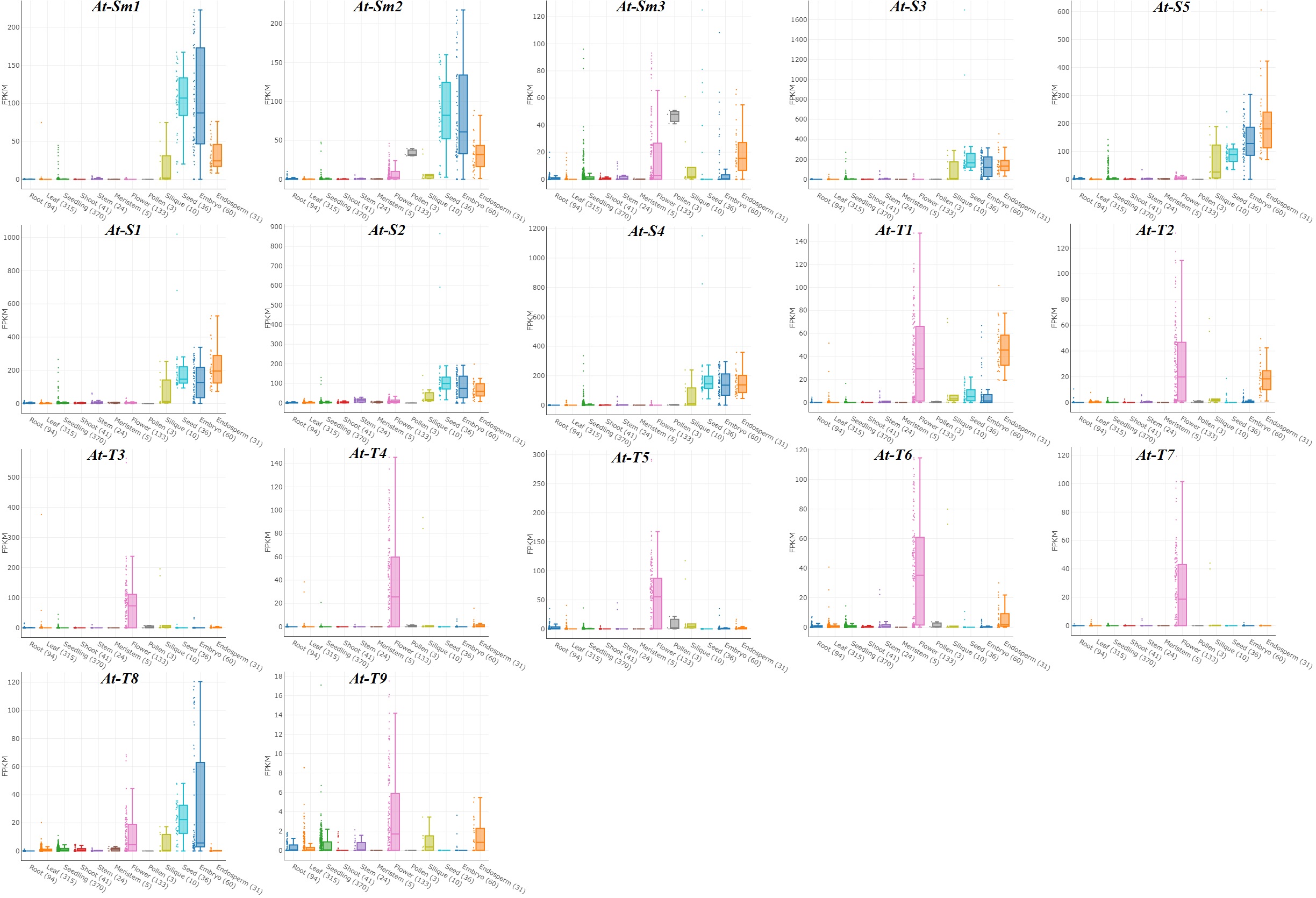

Supplement: Supplementary file 1 [file plants-13-00280-s001.zip › Figure S5.jpg]

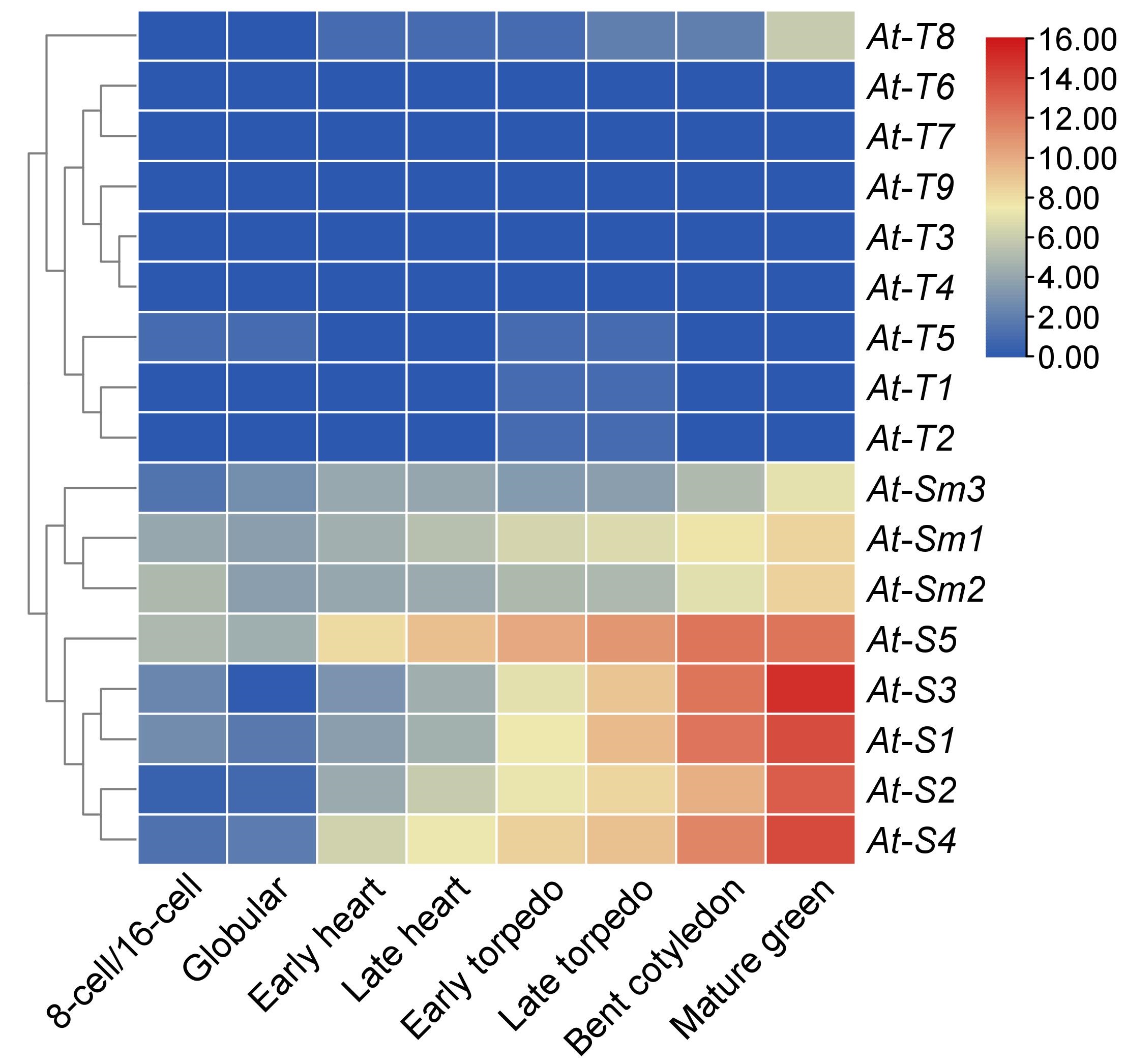

Supplement: Supplementary file 1 [file plants-13-00280-s001.zip › Figure S6.jpg]
